# SF-Assemblin genes in *Paramecium*: phylogeny and phenotypes of RNAi silencing on the ciliary-striated rootlets and surface organization

**DOI:** 10.1186/s13630-019-0062-y

**Published:** 2019-10-29

**Authors:** Ashikun Nabi, Junji Yano, Megan S. Valentine, Tyler Picariello, Judith L. Van Houten

**Affiliations:** 1 University of Vermont, Burlington, VT 05405 USA; 2 State University of New York at Plattsburgh, Plattsburgh, NY 12901 USA; 3 University of Massachusetts Medical School, Worcester, MA 01655 USA; 4 Present Address: Molecular, Cellular and Developmental Biology, Yale University, New Haven, CT 06511 USA

**Keywords:** Cilia, Basal body, Striated rootlet, RNAi

## Abstract

**Background:**

Cilia emanate from basal bodies just underneath the cell membrane. Basal bodies must withstand torque from the ciliary beat and be appropriately spaced for cilia to beat in metachronal waves. Basal body rootlets provide stability for motile cilia. *Paramecium* has three. Our focus is on the largest one, the striated rootlet (SR). *Paramecium* basal bodies align in straight rows. Previously we found a potential role for the SR in this alignment. Here we present a phylogeny of the *Paramecium* homologs of the *SF*-*Assemblin* gene of the SR of *Chlamydomonas,* and the organization of these genes. We describe the phenotypes from RNA interference (RNAi) silencing of genes and gene groups.

**Methods:**

Phenotypes of the RNAi depletions were characterized by immunofluorescence (IF), electron microscopy, and mass spectrometry.

**Results:**

We found 30 genes for *Paramecium* SF-Assemblin homologs (*SFA*) organized into 13 Paralog Groups (further categorized in five Structural Groups). Representatives of Paralog Groups were found in the SRs. Silencing the transcripts of any of the Structural Groups correlates with misaligned rows of basal bodies, SRs, and cortical units. The silencing of Structural Groups was key and gave us the ability to systematically disrupt SR structures and cell surface organization.

**Conclusions:**

Silencing of *SFA* genes and Paralog Groups shows no effects on the SR or the cell surface organization. Silencing of the larger Structural Groups has an enormous impact on rows of basal bodies, SRs and cortical units, and SR striations, and length. Misaligned basal bodies have cilia causing the cells to swim in abnormal paths.

## Background

Cilia are the slender organelles that project from the surface of eukaryotic cells, found in all extant eukaryotes [1]. We focus here on motile cilia, which allow cells like *Paramecium* to swim in their watery environment of a pond or stream and also to sense and respond to their environment [2]. These cilia share many proteins across phyla, which is why the green algae, *Chlamydomonas reinhardtii*, can be used as a model system for cilia development [3, 4]. Similarly, *Paramecium tetraurelia*, long studied for its ciliary structure, beat, and electrical control of this beating, serves as a model system for motile cilia including those of multiciliated cells [5, 6].

At the base of each cilium is the basal body, a modified centriole, with its associated rootlets [7]. While there can be microtubule-based appendages at the basal body, there usually is at least one striated rootlet (SR) composed of proteins unrelated to tubulin. This SR (also known as a kinetodesmal fiber, KF in protists) links the cilium to the cell body. This rootlet has been found to be important for basal body cohesion, anchoring of the basal body, mechanosensation, and chemosensation of sensory neurons of *Drosophila* [8, 9]; long-term stability of photoreceptors by modulating the successful delivery of cargo through IFT particles to the cilium of *Caenorhabditis elegans* [10]; prevention of degeneration of the photoreceptors in mouse and *Drosophila* through physically protecting the thin bridge between the cell body and large light-sensing organelle [11]; and securing the *Tetrahymena* basal body to resist hydrodynamic forces as the cilia beat [12, 13].

**Fig. 1:**
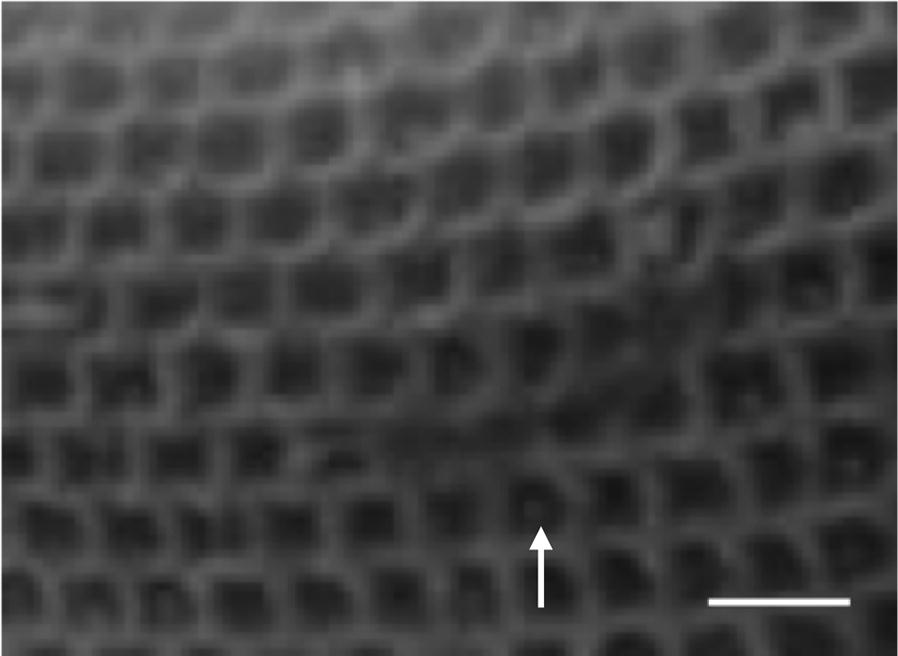
Section of a scanning electron micrograph of a deciliated *P. tetraurelia* cell showing the cortical units that cover the cell surface. Rows of cortical units run between the anterior and posterior poles. One or two basal bodies are in each unit but cannot be seen here. The small structures (arrow) in some of the units are stubs of cilia, which break off at the transition zone during deciliation. Anterior is to the left. Scale bar is 4 μm

The *Paramecium* surface with a thousand or more cilia is organized into roughly rectangular units bounded by ridges and with one or two cilia arising from the depression between the ridges. Figure 1 shows a section from an image of a cell that has been deciliated to better visualize the surface cortical unit pattern. (The little nubs in some of the units are the stubs of cilia that were broken off by trituration to deciliate the cell.) These units align in rows running between the posterior and anterior poles of the cell [14]. This organization keeps the motile cilia beating with their power stroke toward the posterior for efficient swimming. The separation of cilia into cortical units likely is the key to achieving the optimal distance between cilia and orientation of the cilia for metachrony [15].

Our initial evidence for implication of SRs in surface organization came from RNA interference (RNAi) silencing of the human ciliopathy gene Meckelin (*MKS3*) in *Paramecium* that caused the pattern of surface units and ciliary orientation to break down. Rows of basal bodies became disoriented, surface units were misshapen and the SR of the basal body meandered under the surface [16]. A contemporaneous study of *Tetrahymena* showed that the SR and associated proteins secure the basal body to the cell surface to resist hydrodynamic forces as the cilia beat [12, 13]. A breakdown in this resistance led to meandering rows of basal bodies and disrupted surface. These discoveries encouraged us to investigate the *Paramecium* SR further for maintaining the organization of basal bodies and cortical units in rows.

In *Paramecium*, the SR projects from the basal body toward the anterior of the cell past several more anterior basal bodies. The structure comprises proteins with molecular mass ranging from 30 to 36 kDa, some of which are phosphoproteins [17, 18]. These proteins form the very long, striated structure (SR) that is dynamic (i.e., changes length during the cell cycle) [19, 20]. Basal bodies and associated rootlets are embedded into the infraciliary lattice (ICL), a mesh that sits beneath the plasma membrane of *Paramecium*.

Among the best characterized SRs are those of *Chlamydomonas reinhardtii* [21]. This led us to use the gene for *SF*-*Assemblin*, a component of one of the two types of SRs in *Chlamydomonas* [22], to search the ParameciumDB. We began this study by identifying the *SF*-*Assemblin* (*SFA*) genes in the *Paramecium* annotated genome and reconstructing a phylogenetic tree [23]. We organized 30 genes into 13 Paralog Groups and, more importantly, into five Structural Groups based on their primary and secondary amino acid structures, especially the number and location of coiled-coil domains. The identification of Structural Groups was the breakthrough that allowed us to use RNAi to reliably and systematically disrupt SRs. Here we describe the phenotypes of these depletions.

## Materials and methods

### Stock, culture, and chemicals

Cells (stock 51s *P. tetraurelia*, sensitive to killer) were grown in wheat grass medium (Pines International, Lawrence, KS, USA) inoculated with *Aerobacter aerogenes* [24]. All chemicals were purchased from Sigma-Aldrich (St Louis, MO, USA) unless otherwise noted.

### *SFA*s sequence analysis

We used the SF-Assemblin protein sequence from *Chlamydomonas reinhardtii* (Accession number: EDP05674.1) to search for homologous SF-Assemblin protein sequences in the *Paramecium* annotated genome in the dedicated database ParameciumDB (http://paramecium.cgm.cnrs-gif.fr/). All possible protein sequences were checked in the NCBI conserved domain search and the Pfam database (http://pfam.xfam.org/) for the presence of conserved domains of SF-Assemblin protein. Coiled-coil domains were identified by the program SMART [25] and COILS [26]. Finally, the phylogenetic relationships among all the *SFA* genes (nucleotide sequence) were analyzed using the MEGA6 software [27]. See Table 1 for a summary of the *SFA* genes. See Additional file 2: Table S3 for Accession numbers.

**Table 1:** Summary of *Paramecium SF*-*Assemblin* gene terminology

Gene name	Paralog Group	Structural Group
*SFA1a*	Group 1	Structural Group 1
*SFA1b*		
*SFA7a*	Group 7	Structural Group 2
*SFA7b*		
*SFA8a*	Group 8	Structural Group 3
*SFA8b*		
*SFA9*	Group 9—1 member	
*SFA10a*	Group 10	
*SFA10b*		
*SFA10c*		
*SFA10d*		
*SFA11a*	Group 11	Structural Group 4
*SFA11b*		
*SFA12a*	Group 12	
*SFA12b*		
*SFA12c*		
*SFA12d*		
*SFA13a*	Group 13	
*SFA13b*		
*SFA13c*		
*SFA13d*		
*SFA2*	Group 2—1 member	Structural Group 5
*SFA3*	Group 3—1 member	
*SFA4*	Group 4—1 member	
*SFA5a*	Group 5	
*SFA5b*		
*SFA6a*	Group 6	
*SFA6b*		
*SFA6c*		
*SFA6d*		

We found all 30 individual *SFA* genes in the *Paramecium* resource for Expressed Sequence Tags (ESTs) in ParameciumDB. They were found to be expressed in vegetative cells but not during cell division.

### RNAi constructs

We designed RNAi constructs for 24 of the 30 *SFA* genes (*SFA1a*, *SFA1b*, *SFA2*, *SFA3*, *SFA4*, *SFA5a*, *SFA5b*, *SFA6a*, *SFA6b*, *SFA6d*, *SFA7a*, *SFA8a*, *SFA8b*, *SFA9*, *SFA10a*, *SFA10d*, *SFA11a*, *SFA11b*, *SFA12a*, *SFA12b*, *SFA12c*, *SFA13a*, *SFA13c* and *SFA13d*). Only 24 RNAi constructs for these genes were necessary because some constructs silenced more than one gene. Constructs were designed from the sequences in the *Paramecium* annotated genome using the ParameciumDB database. Gene ID numbers in ParameciumDB and gene nucleotide base positions for RNAi constructs are available in Additional file 2: Table S1. In order to design specific constructs, we carried out off-target analysis for each construct (http://paramecium.cgm.cnrsgif.fr/cgi/alignment/off-target.cgi). Specific oligonucleotide primers (Additional file 2: Table S2) were used to amplify the designed sequence by using Genomic DNA as a template. The degree to which we were successful in targeting specific genes for silencing was verified through RT-PCR (see below).

Genomic DNA was purified by organic extraction as described in [16]. PCR amplicons were then cloned directly into pCR2.1-TOPO vector (Invitrogen/Life Technologies), transformed into bacteria and sequenced according to the manufacturer (Invitrogen/Life Technologies) instructions. Correct insert sequences were cut from the pCR2.1-TOPO vector and ligated into the double T7-promoter vector L4440 (Addgene, Cambridge, MA, USA) using the Quick Ligation™ reaction kit (New England BioLab Inc, Ipswich, MA, USA) as per the kit instructions. All vector inserts were sequenced. All RNAi plasmid constructs were maintained in *Escherichia coli* DH5α bacteria at − 80 °C in a 30% glycerol stock.

### RNAi feeding

*Escherichia coli* strain HT115 (DE), which lacks RNaseIII, was used for RNAi feeding. HT115 bacteria were transformed with 50 ng of RNAi plasmid constructs of interest. HT115 bacteria transformed with L4440 with no insert were used as control. Overnight cultures of HT115 bacteria transformed with an *SFA* RNAi construct or control plasmid were used to inoculate 50 mL of Luria broth containing ampicillin (100 µg/mL) (LB-AMP). The bacterial cultures were incubated at 37 °C in a shaking incubator (New Brunswick Scientific) until they reached 595 nm optical densities (OD) of 0.3–0.4. At the desirable OD, cultures were induced with isopropylthio-β-galactoside (IPTG) (RPI Corp., Mt. Prospect, IL, USA) by adding to a final concentration of 125 µg/mL. Cultures were then incubated at 37 °C in a shaking incubator for additional 4 h for double stranded RNA synthesis. The induced bacteria cultures were next centrifuged at 3439×*g* for 10 min at 4 °C (Beckman Coulter, Brea, CA, USA) and the bacterial pellets were resuspended in 100 mL of wheat grass media containing additional stigmasterol (8 µg/mL), ampicillin (100 µg/mL) and IPTG (125 µg/mL). *Paramecium* cells which had recently undergone autogamy (checked by Dippell staining) [28] were washed in Dryl’s solution (1 mM Na_2_HPO_4_, 1 mM NaH_2_PO_4_, 1.5 mM CaCl_2_, 2 mM Na-citrate; pH 6.8). For washing the paramecia, cells were harvested by centrifugation (500×*g*, Damon IEC Division Clinical Centrifuge, Needham Heights, MA, USA), resuspended in Dryl’s solution and again harvested by centrifugation (500×*g*). Approximately 200–300 washed paramecia cells were added to the induced bacterial culture. All RNAi cultures of paramecia and bacteria were maintained at room temperature. Additional amounts of stigmasterol (8 µg/mL), ampicillin (100 µg/mL) and IPTG (125 µg/mL) were added at 24 h and 48 h. Paramecia were collected by centrifugation (500×*g*) at 72 h after feeding (i.e., mixing with the bacteria) and subjected to further analysis unless noted otherwise. All the RNAi experiments were repeated a minimum of three times.

Modifications were made for large scale RNAi to treat large numbers of cells in 6 L of culture. The bacterial cells were induced as above in 500 mL of LB-AMP with IPTG (125 µg/mL) and the bacterial pellets resuspended in 1.5 L of regular wheat grass medium containing stigmasterol (8 µg/mL), ampicillin (100 µg/mL) and IPTG (125 µg/mL). Approximately 6000 paramecia cells were added to the induced bacterial cultures. Additional amounts of induced bacteria (500 mL), ampicillin (100 µg/mL) and IPTG (125 µg/mL) were added to the RNAi cultures at 24, 48 and 72 h. Finally, paramecia cells were collected by centrifugation (500×*g*) at 96 h of RNAi feeding for the isolation of the SRs, immunofluorescence and the extraction of m-RNA.

### Reverse transcription-polymerase chain reaction (RT-PCR)

We used RT-PCR to check the efficacy and specificity of the RNAi feeding procedure following the protocol published previously [29]. A pair of forward and reverse *calmodulin* (*Cam1*) gene primers (Additional file 2: Table S2) was used in RT-PCR as control for the amount of input template and as a check on the consistency of the method. Three different concentrations (diluted tenfold, 100-fold and, if necessary 500-fold) of cDNA were used as template because viewing the results of a dilution series made it easier to compare among conditions. The results, while not quantitative, were highly reproducible, giving confidence that the target mRNA was depleted. All experiments with their accompanying RT-PCR were carried out three times.

The RNAi construct designs were very crucial. For example, since nine Paralog Groups among thirteen have more than one gene, RNAi experiments were designed to deplete the messages of all the genes within a targeted Paralog Group but not affect the messages of other nontargeted Paralog Groups. Even for the four Paralog Groups that have only one gene (*SFA2*, *SFA3*, *SFA4* and *SFA9*), we used RT-PCR to confirm the level of message depletion within a targeted Paralog Group without affecting the nontargeted Paralog Groups.

Analysis of RT-PCR data from various RNAi experiments clearly demonstrates our designed RNAi constructs only depleted the message for the targeted Paralog Group or Structural Group without affecting the message of the nontargeted Groups. Additional file 1: Fig. S1 is a typical RT-PCR result. This figure shows that the target cDNA is greatly reduced without affecting levels of nontarget *SFA* gene cDNA and an unrelated gene, the *calmodulin* (*Cam1*) gene. The image is from a single experiment that is one of the triplicate experiments . Separate controls are carried out for each experiment. The image is unaltered except cropped at the top and bottom.

### Cell immunofluorescence

Paramecia cells in 100 mL cultures were fed bacteria for control or RNAi as described above. After the RNAi feeding, the cultures were divided in half for immunofluorescence as we described previously [16, 30] and for RT-PCR as we describe above. In this way, we could correlate the phenotype of RNAi with mRNA.

For the visualization of the SR structures and basal bodies, primary antibodies were as follows: rabbit anti-SR at a dilution of 1:400 ([17] a gift from Janine Beisson, Centre de Génétique Moléculaire, Gif-sur-Yvette, France) and mouse anti-detyrosinated α-tubulin mouse monoclonal antibody (ID5) at a dilution of 1:500 (Synaptic Systems, Göttingen, Germany). For the visualization of ridges of cortical units with their SRs, primary antibodies were as follows: mouse 2F12 at a dilution of 1:200 [31] gift from Jean Cohen, Gif-sur-Yvette, France) and rabbit anti-SR at a dilution of 1:400. All the images were captured using the DeltaVision^®^ restoration microscopy system (Applied Precision), consisting of an inverted Olympus IX70 microscope (Olympus America, Center Valley, PA, USA) and a Kodak CH350E camera (Rochester, NY, USA).

For the visualization of the basal bodies along with all three rootlets, tubulin and the basal bodies had to be visualized with the same fluorophore on the secondary antibodies. Therefore, we deciliated cells just before immunostaining since one of the antibodies (anti-α tubulin) stains the microtubule-based rootlets as well as cilia, which interfere with visualization of rootlets. Cells were deciliated as previously described [30]. Primary antibodies for the immunostaining of deciliated cells were as follows: rabbit anti-SR at a dilution of 1:400, mouse ID5 at a dilution of 1:500 (Synaptic Systems, Göttingen, Germany) and mouse anti-α-tubulin at a dilution of 1:200 (Sigma-Aldrich, St Louis, MO, USA).

### Plasmid injection

We used N-terminal FLAG pPXV plasmid (modified plasmid, courtesy of Dr. W. John Haynes, University of Wisconsin, Madison, WI, USA) to clone 13 different *SFA* genes (*SFA1a*, *SFA2*, *SFA3*, *SFA4*, *SFA5a*, *SFA6b*, *SFA7a*, *SFA8a*, *SFA9*, *SFA10a*, *SFA11a*, *SFA12a* and *SFA13a*). All the gene sequences are available in ParameciumDB (http://paramecium.cgm.cnrs-gif.fr/). Accession numbers are available in Additional file 2: Table S1. All the primers used for FLAG-epitope tagging are listed in Additional file 2: Table S2. The target gene sequence was amplified by using Q5TM Hot Start high fidelity DNA polymerase (New England BioLab Inc, Ipswich, MA, USA) according to the manufacturer’s protocol. Resulting amplicons were inserted into the pPXV plasmid using restriction enzymes (*Nhe*I/*Kpn*I or *Apa*I/*Sac*I) (New England BioLab Inc, Ipswich, MA, USA) and the amplicon sequences were confirmed by sequencing. All the FLAG pPXV plasmids containing the target gene sequences were linearized by using *Not*I restriction enzyme (New England BioLab Inc, Ipswich, MA, USA) and injected as previously described [30]. The presence of pPXV-3XFLAG-SFA in individual clones was confirmed using PCR with extracted genomic DNA as a template.

### Localization study

For the localization study of SFA proteins, individual clones expressing FLAG-SFAs were grown in 50 mL wheat grass culture media at 22 °C for 48 h. Cells were immunostained and imaged as described above. Primary antibodies for the immunostaining were as follows: for FLAG-SFA we used mouse anti-FLAG M2 clone at a dilution of 1:300 (Sigma-Aldrich, St Louis, MO, USA) and for basal bodies we used rabbit anti-centrin (anti-*Tetrahymena* basal body centrin, gift from Mark Winey and Alex Stemm-Wolf, University of Calif. Davis) at a dilution of 1:1000. All the images were captured using the same microscope system as described before. We followed the same procedure for 13 representative SFA proteins.

### Cell fractionation

We followed the protocol published by [17, 18] with some modifications, primarily the use of Optiprep instead of Percoll gradients. Cells were harvested from 6 L of culture fluid (4000–6000 cells/mL) by continuous flow centrifugation (IEC clinical centrifuge, 300×*g*) and washed three times in TEK buffer (20 mM Tris, 5 mM EGTA, 100 mM KCl; pH7) by resuspending the cells in 100 mL of TEK buffer followed by centrifugation (500×*g*). Cell cortices were prepared as previously published (see above). The final pellet was resuspended in 600 µL PHEM buffer (60 mM PIPES, 25 mM HEPES, 10 mM EGTA, 2 mM MgCl_2_, pH 6.9) and layered on a PHEM-Optiprep density gradient made of steps of 40%, 35%, 30%, 25%, 20%, 15% and 0% Optiprep (500 µL of each) in PHEM buffer. The gradient was centrifuged in Beckman Coulter ultracentrifuge at 45,000 rpm (SW60 Ti rotor) for 2 h. After centrifugation, each PHEM-Optiprep layer in the gradient was collected separately and diluted tenfold with membrane buffer (10 mM Tris–Cl, 10 mM Tris-base, 50 mM KCl, 5 mM MgCl_2_, 1 mM EGTA, pH 7.4). To remove the Optiprep from the proteins, each suspension was centrifuged at 48,750×*g* (in Beckman J2-21) for 30 min. Each pellet was resuspended in 100 µL PHEM buffer.

To examine PHEM-Optiprep layers for SR structures, we used cells expressing FLAG epitope tagged *SFA8a*, *SFA7a* or *SFA2* genes. The PHEM-Optiprep fractions from the preparation of these cells were mixed 1:1 with 2% low melting agarose (Sigma-Aldrich, St Louis, MO, USA) in PHEM. Agarose gel pieces were fixed for 30–40 min with fixation buffer. (See above immunofluorescence protocol.) Primary antibody staining using anti-FLAG antibody (anti-FLAG M2 clone at a dilution of 1:300; Sigma-Aldrich, St Louis, MO, USA) was followed by washing and secondary antibody staining (Alexa Fluor 555 goat anti-mouse; Molecular Probes/Invitrogen, Grand Island, NY, USA). All the buffers for primary antibody staining, secondary antibody staining, washing, and image acquisition were the same as described above in the cell immunofluorescence protocol.

### Negative staining and transmission electron microscopy (TEM)

Delaware Biotechnology Institute carried out negative staining (protocol kindly provided by Chad Pearson) and TEM of the fraction from the Optiprep preparation in which the immunofluorescent structures were found. In brief, Carbon-coated 400 mesh copper grids were rendered hydrophilic with a PELCO easiGlow Glow Discharge Cleaning System. The grids were floated on drops of sample for several seconds, washed on four drops of water and then negative stained with 2% uranyl acetate (aq). After drying, samples were examined with a Zeiss Libra 120 transmission electron microscope operating at 120 kV. Images were acquired with a Gatan Ultrascan 1000 CCD camera. The grids were then stored in a grid box for imaging. Although the images provided by the Delaware Biotechnology Institute generally showed one structure at a time including a scale bar, we analyzed 20 SRs or more for each experiment of control or depleted cells. All experiments were repeated three times for a total of 60 SRs analyzed per condition.

### Mass spectrometry analysis

Proteins of the 30% Optiprep fraction were separated on a gradient (5–18%) SDS–polyacrylamide gel electrophoresis (PAGE) gel. The gel was systematically cut into sections and prepared for mass spectrometry as described before [30]. The prepared sections were dissolved in 7 µL 0.1% formic acid and 2.5% acetonitrile, and 2 µL were analyzed on the Thermo Q-Exactive mass spectrometer coupled to an EASY-nLC system (Thermo Fisher). Peptides were separated on a fused silica capillary (12 cm × 100 μm I.D) packed with Halo C18 (2.7 μm particle size, 90 nm pore size, Michrom Bioresources) at a flow rate of 300 nL/min. Peptides were introduced into the mass spectrometer via a nanospray ionization source at a spray voltage of 2.2 kV. Mass spectrometry data were acquired in a data-dependent top-10 mode, and the lock mass function was activated (*m*/*z*, 371.1012). Full scans were acquired from *m*/*z* 350 to 1600 at 70,000 resolutions (automatic gain control (AGC) target, 1e6; maximum ion time (max IT), 100 ms; profile mode). Resolution for dd-MS2 spectra was set to 17,500 (AGC target: 1e5) with a maximum ion injection time of 50 ms. The normalized collision energy was 27 eV. A gradient of 0 to 40% acetonitrile (0.1% FA) over 55 min was applied. The spectra were searched against the Ptetraurelia_peptides_v1.99.13 protein database (http://paramecium.cgm.cnrs-gif.fr/download/fasta/) by Proteome Discoverer (PD) 1.4. The search parameters permitted a 10 ppm precursor MS tolerance and a 0.02 Da MS/MS tolerance. Carboxymethylation of cysteines was set up as fixed modifications and oxidation of methionine (M) was allowed as variable modifications. Up to three missed tryptic cleavages of peptides were considered with the false-discovery rate set to 1% at the peptide level.

### Swimming behavior analysis

Swimming behavior was analyzed using darkfield microscopy. The Control, Paralog Group-depleted, or Structural Group-depleted cells were harvested by centrifugation after 72 h of feeding (500×*g*, Damon IEC Division Clinical Centrifuge, Needham Heights, MA, USA). Harvested cells were resuspended in 5 mM KCl buffer (1 mM citric acid, 1 mM Ca(OH)_2_, and 1 mM Tris-Base, pH 7.02 with Tris-Base). Cells in 5 mM KCl buffer were placed in a small drop on a slide on a dissecting microscope stage. Another drop of 5 mM KCl buffer was placed near the drop containing the cells. A connection was made between the two drops using a Pasteur pipette. Swimming trajectories of the cells were captured as darkfield images of tracks of swimming *Paramecium* cells using a Canon DS digital camera mounted on the dissecting microscope (Bausch and Lomb) with an exposure time of 2 s.

## Results

### *SF*-*Assemblin* (*SFA*) genes in *Paramecium tetraurelia*

Using the gene for *SF*-*Assemblin,* a component of one of the two types of SRs in *Chlamydomonas* [22], to search the ParameciumDB, we identified thirty *Paramecium tetraurelia* genes. When translated from the nucleic acid sequences, their proteins have the characteristic domains of the *Chlamydomonas* SF-Assemblin protein (N-terminal head domain and C-terminal rod domain) as confirmed by the NCBI conserved domain search and Pfam database. We call them *SFA* genes and we later show that they are expressed in the *Paramecium* SR. We make no claim that these are the only proteins of the SR, but rather they are *SF*-*Assemblin* homologs in the SR.

**Fig. 2:**
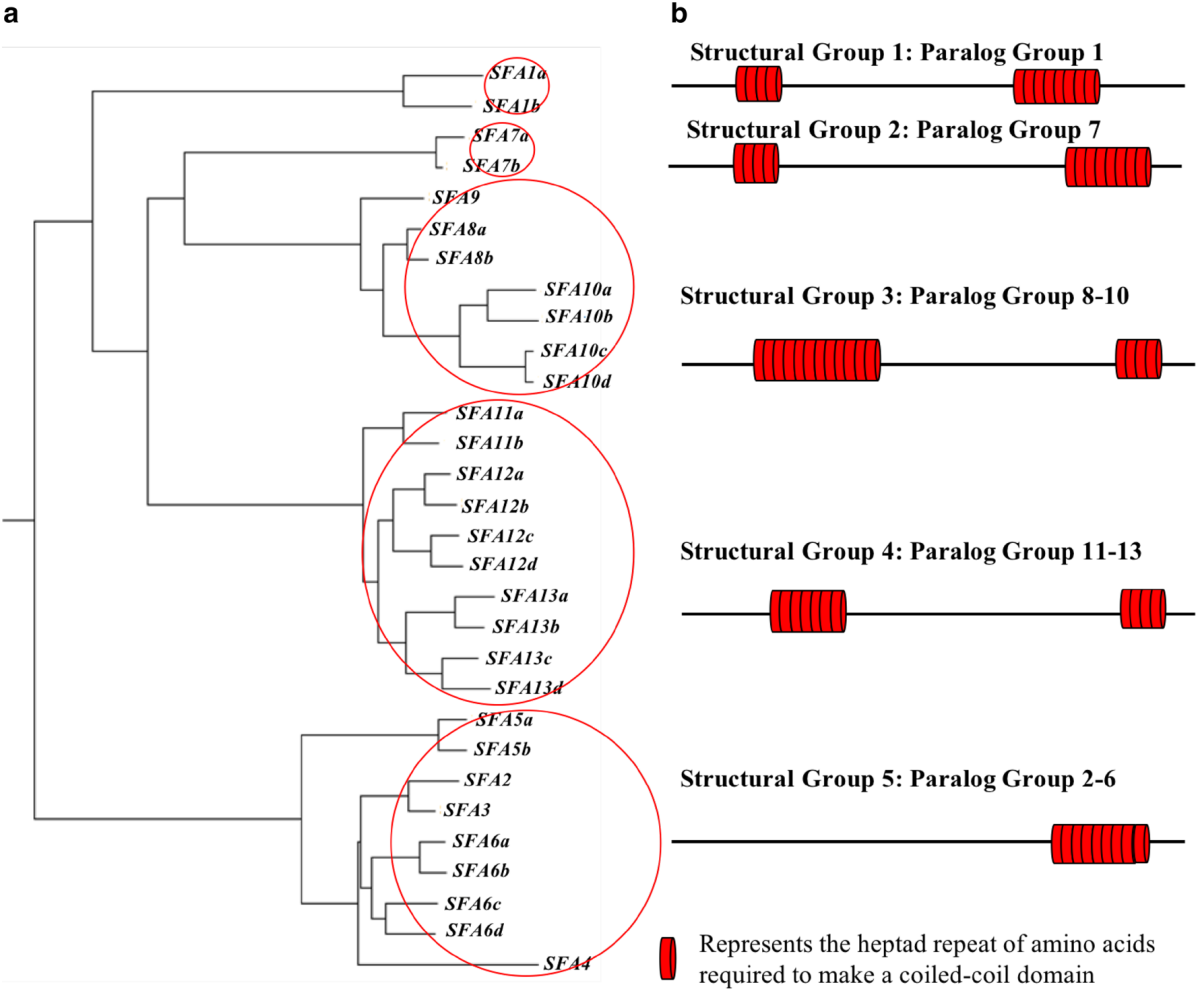
The phylogenetic relationships among the *SFA* genes in *Paramecium*. Red circles in **a** show the five Structural Groups based on their potential for and locations of coiled-coil domains. Each red circle marks the members of a Structural Group. **b** The positions of the coiled-coil domains in the five Structural Groups

Evolutionary analyses of the 30 genes were conducted in MEGA6 [27] (Fig. 2a). The evolutionary relationships were inferred using the Neighbor-Joining method [32]. The MEGA6 software uses the Maximum Composite Likelihood method to measure evolutionary distances in the units of base substitutions per site. The analysis involved all 30 nucleotide sequences. The final dataset includes 715 positions and eliminates all positions having the gap and missing data sets.

The analyses of the *SF*-*Assemblin* homologs’ nucleic acid and amino acid sequences show several layers of organization. The 30 *SFA* genes fall into 13 Paralog Groups. We simplified the nomenclature from *SF*-*Assemblin SR* to *SFA* for a gene name. *SFA* is distinguished from a Paralog Group in Fig. 2 and Table 1. We use the term Paralog Group for groupings of genes from the whole genome duplications. For example, genes *SFA1a* and *SFA1b* are results of one of the three whole genome duplications and could be called ohnologs. Together these genes form Paralog Group 1. In another example, *SFA8a* and *SFA8b* form Paralog Group 8; *SFA10a, SFA10b, SFA10c* and *SFA10d* form Paralog Group 10. *SFA9* is the single gene in its Paralog Group 9. For clarity, we use *SFA* in the gene name as above and SFA in the protein name, but drop “SFA” from the Paralog and Structural Group names.

We further organized the 13 Paralog Groups into five Structural Groups based on sequences (in Fig. 2b, see red circles around the Structural Groups) and their hypothetical primary and secondary protein structures. The SF-Assemblin of *Chlamydomonas* is characterized by coiled-coil domains that facilitate the head-to-tail interactions underlying the large macromolecular structure of this rootlet [21]. When we examined the hypothetical translation products of each *Paramecium SFA* gene, we found that the gene members of each Structural Group shared the predicted number and location of coiled-coil domains and the number of heptamers that make up each coiled-coil domain (Fig. 2b). Structural Groups 1 and 2 each have two members whereas Structural Groups 3, 4 and 5 have seven, ten and nine gene members, respectively (Table 1).

Four of the Structural Groups have genes that code for proteins with two predicted coiled-coil domains. The exception is Structural Group 5, which has genes with one predicted coiled-coil domain. These domains were identified by the program SMART [25] and COILS [26]. The position and length of the coiled-coil domains in the SFA proteins within a particular Structural Group are similar but vary considerably between Structural Groups (Additional file 2: Table S3). The amino acid sequence alignments of coiled-coil domains from Structural Groups also showed that the amino acid sequences of the coiled-coil domains of SFA proteins within a Structural Group are very similar, but sequences are not similar between Structural Groups (data now shown).

We believe that our identification of *SF*-*Assemblin* homologs in the ParameciumDB is exhaustive because we found five more sequences than had previously been labeled as “kd” for kinetodesmal fiber genes. We also found some genes previously labeled in the data base as “kd” but that do not code for the characteristic domains of *Chlamydomonas* SF-Assemblin protein. We named these genes *SR*-*like* (*SRL*). We used MEGA6 software to construct a phylogenetic tree, which includes both *SFA* and *SRL* nucleotide sequences (Additional file 1: Fig. S2). The nucleotide sequences of the *SRL* genes (sequences that are available in ParameciumDB and used for phylogenetic analysis) are very different from the *SFA* genes based on the size and nucleotide sequence similarity. We found two anomalies in the ParameciumDB that identified two genes that are duplicates of *SFA7a* and *SFA7b* (data not shown). This adds to our confidence that we have identified all *SFA* sequences.

### Epitope tagging shows that Flag-SFA proteins are in the SR

**Fig. 3:**
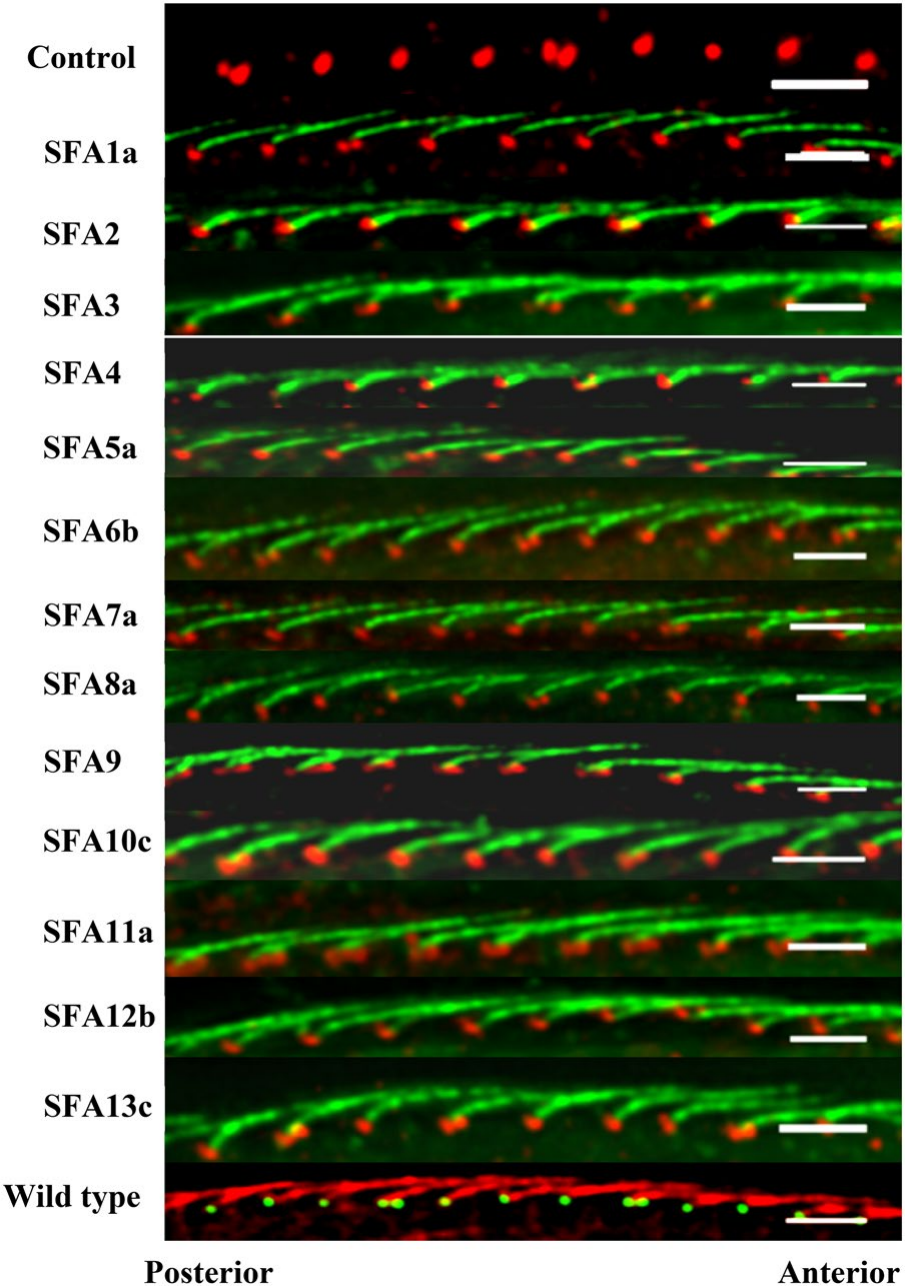
SFA *Paramecium* proteins present in and distributed throughout the SR structure. Cells expressing only the FLAG epitope (control) or a FLAG-SFA representative of a Paralog Group were immunostained with anti-basal body centrin (red) and anti-FLAG (green). Panels show the side-view images of control, SFA1a, SFA2, SFA3, SFA4, SFA5a, SFA6b, SFA7a, SFA8a, SFA9, SFA10c, SFA11a, SFA12b, SFA13c cells expressing FLAG or FLAG-SFA. The basal bodies are red and the SRs are green. An additional control is the image from a wild-type cell with green basal bodies (ID5) and red SRs (anti-SR). Scale bars are 3μm

We randomly selected one gene from each of the 13 Paralog Groups (*SFA1a*, *SFA2*, *SFA3*, *SFA4*, *SFA5a*, *SFA6b*, *SFA7a*, *SFA8a*, *SFA9*, *SFA10c*, *SFA11a*, *SFA12d,* and *SFA13c*) for FLAG epitope tagging to determine whether the gene products were in the SR structures and, if so, where the product was located in the SR. Wild-type *P. tetraurelia* cells injected with the 5′-3×FLAG plasmid to express the FLAG peptide served as control cells. Uninjected wild-type *P. tetraurelia* cells served as a second control. Cells injected with 5′-3×FLAG-*SFA* or 5′-3×FLAG were treated with anti-FLAG and anti-centrin (for basal bodies). The second control uninjected wild-type cells (Fig. 3) were treated with anti-SR and ID5 to visualize the SRs and basal bodies, respectively.

FLAG staining in the cells expressing FLAG-SFA proteins shows that these proteins are present in and distributed throughout the SRs (green) (Fig. 3). It appears, within the limits of the fluorescence microscopy (resolution 200 nm), that the FLAG-SFA proteins are distributed from base to tip. In addition, in all the representative FLAG-SFA-expressing cells, the SRs (green) are similar in appearance to those in the second control (red) nonexpressing wild-type cells.

Proteins without the characteristic domains of the SF-Assemblin that we named SR-like (SRL) do not appear to localize in the SRs. Immunostaining of FLAG-SRL protein expressing cells with anti-FLAG shows that FLAG-SRLs are located intracellularly, in the basal bodies, in the cilia, or in the epiplasm just beneath the cortical unit (Additional file 1: Fig. S3). The epiplasm has a very characteristic cup shape [33] that can be seen clearly in this figure.

### Phenotype 1: Cells depleted of Structural Groups show basal body row misalignment, SR misalignment, and abnormal gross SR appearance

**Fig. 4:**
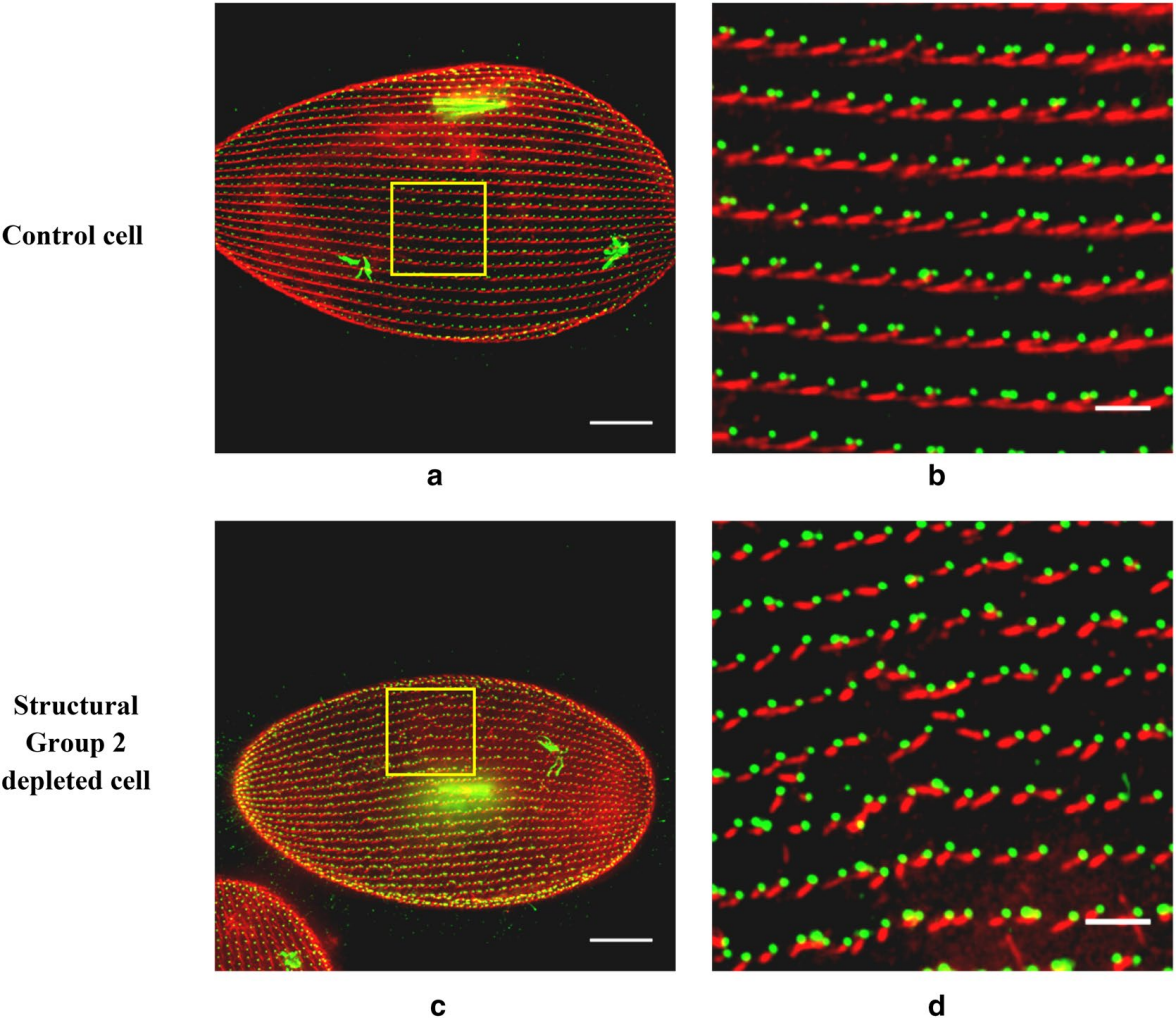
Structural Group depletion that can lead to basal body row misalignment and abnormal SR appearance. **a**, **c** Control and Structural Group 2 (also Paralog Group 7)-depleted cells, respectively. The yellow box in each image is enlarged (**b**, **d**) to highlight basal body rows. The basal bodies are green (ID5 antibody) and SRs are red (anti-SR). **a**, **b** Straight rows of basal bodies as well as SR rows that extend between the posterior pole and the anterior pole. **c**, **d** The severely misaligned basal body rows as well as abnormal SRs (shorter and not pointed toward the anterior pole) and disrupted SR rows. All images are of the dorsal surfaces, but misalignments can occur anywhere on the surface except the oral groove. Scale bars are 15 μm (**a**, **c**) and 3 μm (**b**, **d**)

We began our study of phenotypes by depleting mRNA for the two *SFA* genes *SFA7a/b*, which are almost identical in DNA sequence and can be silenced with the same RNAi construct. The phenotypes of the L4440-fed control cells and the RNAi-fed cells were examined with immunofluorescence (Fig. 4). In all cells, ID5 marks the basal bodies (green) and anti-SR marks the SRs (red). The whole cell images (Fig. 4a, c) comprise the stacks of Z-sections approximately 10 μm thick to ensure all basal bodies along with SRs are visible. In L4440-fed control cells (Fig. 4a), basal bodies and SRs have a highly organized characteristic pattern. Basal bodies are in straight rows from the posterior to the anterior pole on both the dorsal and the ventral surfaces of the cell. The SRs originate from the basal bodies, extend toward the anterior pole of the cell, and traverse several basal body units (Fig. 4b). The test RNAi treated cells (Fig. 4c with inset Fig. 4d) show severely disrupted basal body row alignments, abnormal SR orientation and shorter shape over the cell surface except the oral groove area.

**Fig. 5:**
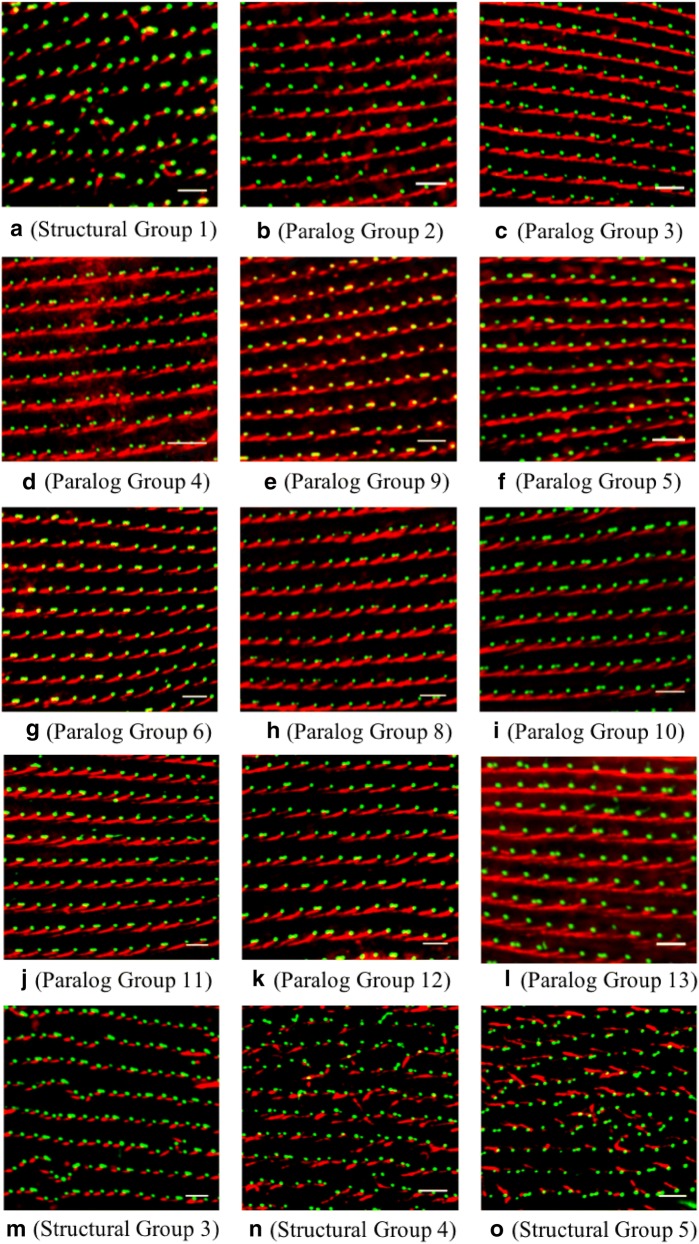
Basal body row alignment and SR appearance in cells with Paralog Group or Structural Group depletion. All images of cell surfaces show basal body rows (ID5 antibody) in green and SRs in red (anti-SR). **a** An image of a cell with Structural Group 1 depleted. **b**–**l** The representative images of cells depleted of Paralog Groups 2–13. **m**–**o** The representative images of the cells depleted of Structural Groups 3–5. Depletion of Paralog Groups (**b**–**l**) does not affect basal body row alignment or the organization of SRs, whereas depletion of Structural Groups (**a**, **m**–**o**) results in misaligned basal body rows and abnormal SR rows. Scale bars are 3 μm. **f**–**h** Have been rotated for easier comparison

When we depleted other *SFA* gene transcripts—we did not find these effects except for *SFA1a/b* (Fig. 5a). The *SFA1a* and *SFA1b* genes are not close enough in sequence to silence both genes with one RNAi construct. Therefore, cells were concurrently fed bacteria with RNAi constructs for both *SFA1a* and *SFA1b*, resulting in a dramatic phenotype of misaligned basal bodies and abnormally oriented and shaped SRs is seen (Fig. 5a).

Table 1 and Fig. 2a show that *SFA1a/b* and *SFA7/b* are the only members of Paralog Groups 1 and 7, respectively. We tested whether silencing each of the 11 other Paralog Groups would produce the same disruptions of the surface. However, none of these RNAi experiments produced the same phenotype as with *SFA1a/b* and *SFA7/b* (Fig. 5b–l). Even depletion of single genes that are the only members of their Paralog Groups (Paralog Groups 2, 3, 4 and 9) produced no obvious phenotypic changes (Fig. 5b–e).

Examination of Table 1 and Fig. 2 showed us that *SFA1a/b* and *SFA7a/b* are the sole members of their respective Paralog Groups 1 and 7, but also, they are *the only members of their Structural Groups 1 and 2.* When transcripts for other Structural Groups are depleted, the extreme phenotype of basal body row and SR disorientation are clear: Structural Group 3 (Fig. 5m), Structural Group 4 (Fig. 5n) or Structural Group 5 (Fig. 5o).

The testing of the Structural Groups’ roles in the surface patterning and SR orientation required many RNAi constructs to concurrently silence all of a Group’s genes and RT-PCR to check on-target specificity. For example, to silence Structural Group 5, nine genes from four Paralog Groups had to be silenced by RNAi. On-target vs off-target depletions of the transcripts from whole Structural Groups or Paralog Groups were confirmed by triplicate RT-PCR. (See Additional file 1: Fig. S1 for an example.)

Note that the normal SR rows resemble cables made of overlapping multiple SRs where they rise to the ridge of the cortical unit and are straight, e.g., Figure 3 Wild type, Figs. 4b, 5b–l. The curvature of the SR as it rises from the basal body to the cortical unit ridge cannot be seen with these images. In comparison, Structural Group-depleted SRs do not fall into a cable-like structure. In Figs. 4 and 5, these SRs appear to be shorter than those of the control. Note also in Fig. 5a, n, and o that the SRs not only leave their rows, but can be directed across or backward. Some show a curly structure. This phenotype will be also seen below where cortical unit rows are misaligned.

### Phenotype 2: Cells depleted of Structural Groups show distorted cortical units

To explore whether the shape and alignment of the cortical units were affected by depletion of Structural Group transcripts, we immunostained cells with 2F12 and anti-SR antibodies which decorate the ridges of the cortical units and SRs, respectively. Normal cortical units are bounded by ridges elevated above the center of the unit with its one or two basal bodies (Fig. 1). The cortical units align with the basal body rows between the posterior and anterior poles of the cell. Each SR originates from the basal body within a cortical unit, extends toward the anterior of the cell and transverses more than one anterior cortical unit. (If there are two basal bodies in the unit, only the posterior basal body has an SR.)

**Fig. 6:**
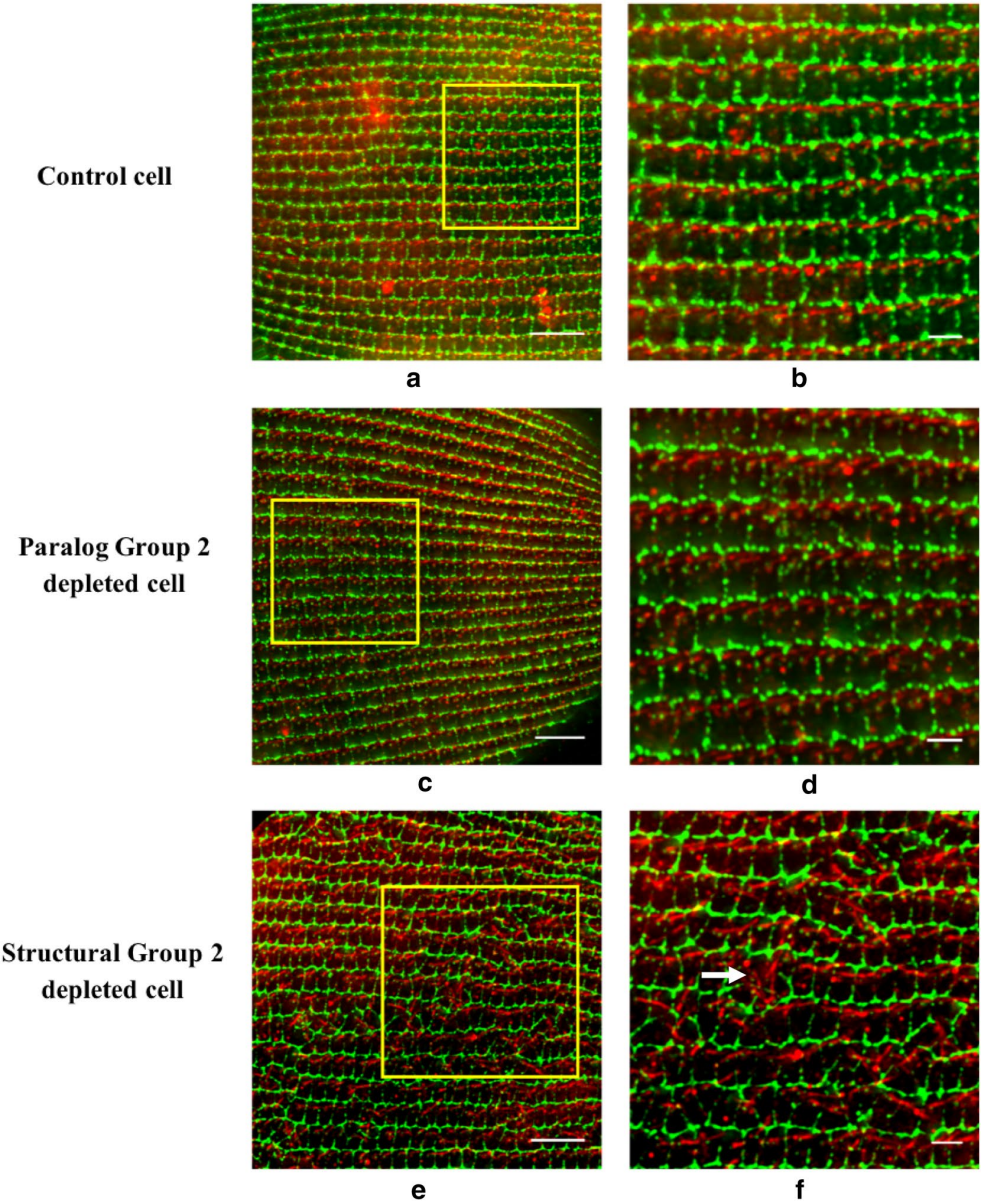
Structural Group depletion causing distorted cortical unit shape as well as abnormal SRs. **a**, **c**, and **e** Control, Paralog Group 2- and Structural Group 2-depleted cells, respectively. The yellow box in each image is enlarged (**b**, **d**, and **e**) to show cortical unit rows (green) with SRs (red). **b**, **d** Organized cortical units as well as SR rows that extend between the posterior pole and the anterior pole in the Control and Paralog Group 2-depleted cells, respectively. **f** The severely distorted cortical units and abnormal SR rows in a Structural Group 2-depleted cell. Arrow in **f** points to SRs that are directed across the row and are abnormally curved in shape. All images are of the dorsal surface, but distortion of cortical units with abnormal SRs in Structural Group-depleted cells can occur anywhere on cells’ surfaces except the oral groove. Antibodies used are anti-Cortical unit ridge antibody (green) and anti-SR (red). Scale bars are 10 μm (**a**, **c,** and **e**) and 3 μm (**b**, **d**, and **f**)

In control L4440-fed cells, cortical units along with SRs are normally organized in a highly ordered pattern on the surface of the cell (Fig. 6a, b). Note the cables of red staining of SRs along the inside of the cortical unit row (Fig. 6a, b). The cells depleted of Paralog Group 2 show the same normal pattern of organization of the cortical units along with the SRs (Fig. 6c, d). We observed same normal phenotype with depletion of transcripts from other Paralog Groups (except the Paralog Groups that themselves forms the Structural Groups, see the following).

In contrast, cells depleted for Structural Group 2 (Table 1) show multiple areas of distorted cortical units with a loss of alignment of these cortical units in straight lines (box in Fig. 6e). Where the cortical units are misaligned, the SRs are also misdirected (box Fig. 6e, f) in contrast to areas of intact cortical rows where the SRs are normally organized (Fig. 6e). The SRs in these cells in the area of distorted cortical units are directed away from the axis of the posterior–anterior poles of the cell (Fig. 6f). Some of these SRs (white arrow Fig. 6f) seem to veer across cortical units and some are wavy in shape.

These abnormalities can be found anywhere on the cell surface except the oral groove. We observed the same dramatic phenotype in cells depleted of other Structural Groups 1, 3, 4, 5 (data not shown).

### Phenotype 3: Cells depleted of structural groups are ciliated and able to swim

**Fig. 7:**
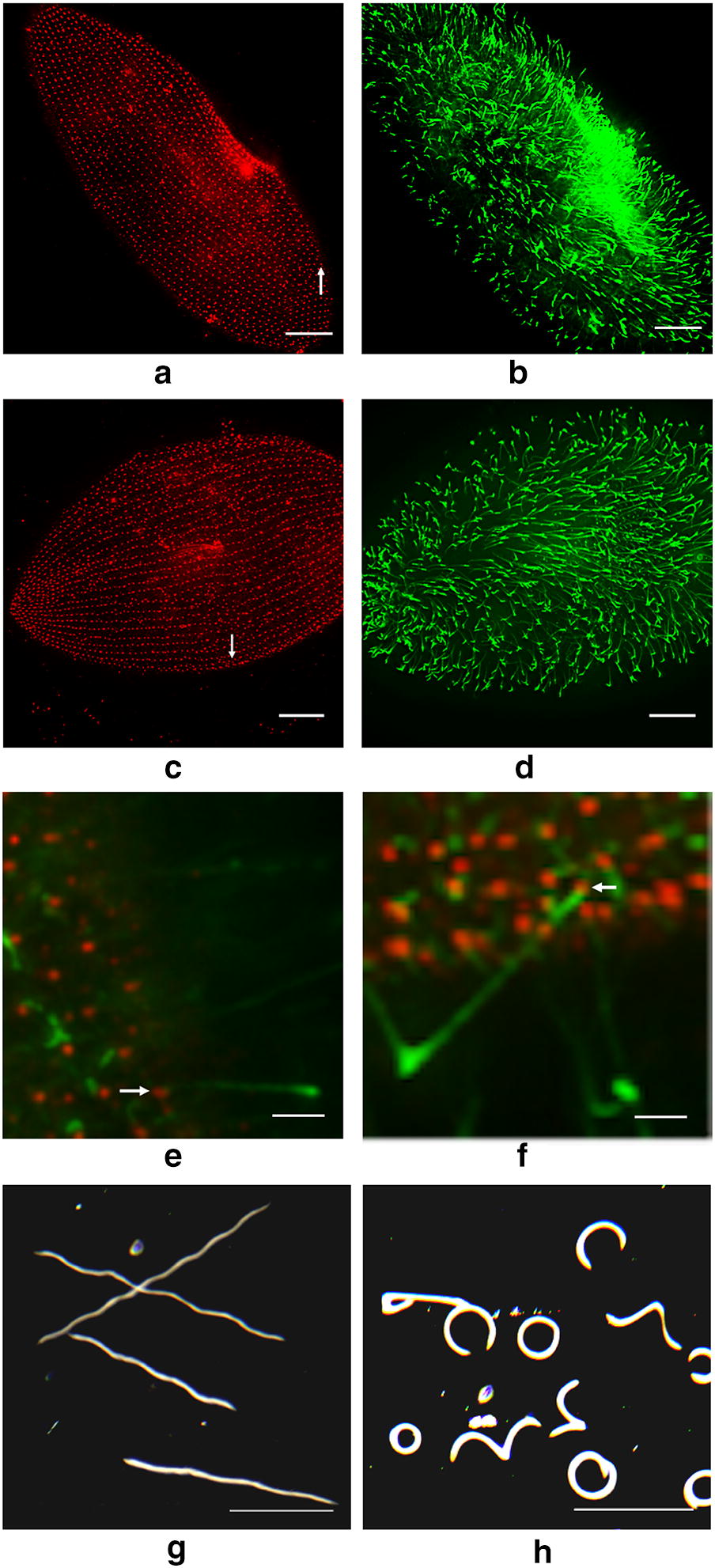
Immunofluorescence images demonstrating that the cells depleted of Structural Groups are ciliated and darkfield images show effects of depletion on swimming behavior. In **a** and **b,** immunofluorescence images of Control cells show straight basal body rows (red) and cilia (green), respectively. In **b** and **d** immunofluorescence images of Structural Group 1-depleted cells show that the cells have cilia (green) and misaligned basal body rows (red). In **a** and **c**, areas pointed out by white arrows are blown up in **e** and **f** showing that cilia emanate from basal bodies of both Control and Structural Group 1-depleted cells, even in an area with misaligned basal bodies. Scale bars are 15 μm (**a**–**d**) and 3 μm (**e**, **f**). **g**, **h** The swimming patterns of cells taken by darkfield microscopy. We show here examples of Control and Structural Group 1-depleted cells. Scale bar is 1 mm

Despite the disruption of rows of basal bodies and cortical units, the cells depleted of a Structural Group are viable and are ciliated. In Fig. 7a, c, note the basal bodies in red in the control and Structural Group-depleted cell, where there is a significant misalignment of basal bodies. Figure 7b, d show that there are many cilia, including in the areas of misalignments (Fig. 7d). An examination of merged enlarged images (Fig. 7e, f) show that cilia emanate from the control and also from misaligned basal bodies, indicating that these basal bodies are docked at the membrane.

Additional evidence of ciliation is that the Structural Group-depleted cells are able to swim, albeit with inefficient and loopy paths compared to controls (Fig. 7g, h). We chose a K buffer in which the cells should swim in long relatively straight paths. The looping paths of the depleted cells were very obviously different. Such paths are expected if the cilia no longer pull with their power stroke in metachronal waves toward the posterior, but instead pull in multiple directions working against each other. The result is much like the proverbial row boat when oars are pulling in opposite directions. If only basal bodies that remain in their straight rows are motile, this phenotype would not occur. Cells would swim slowly forward in straight paths. The observed swimming paths require that some cilia pull against the normal power stroke that should be toward the posterior.

### Phenotype 4: Basal bodies in cells depleted of Structural Group proteins have rootlets emerging at normal angles

Each *Paramecium* basal body connects to three rootlets on particular microtubule triplets that give the basal body a functional asymmetry. Two rootlets are microtubule-based and project toward the posterior pole of the cell (postciliary rootlet-PR) or laterally toward the adjacent rows of basal bodies (transverse rootlet-TR) [14, 20, 34]. The third rootlet is the SR. These three rootlets of the basal body are at fixed angles because they are attached to specific triplet microtubules of the basal body. The SR emanates from the left side of the basal body at triplets 6 and 7, and extends toward the anterior past several more anterior basal bodies [14, 34].

**Fig. 8:**
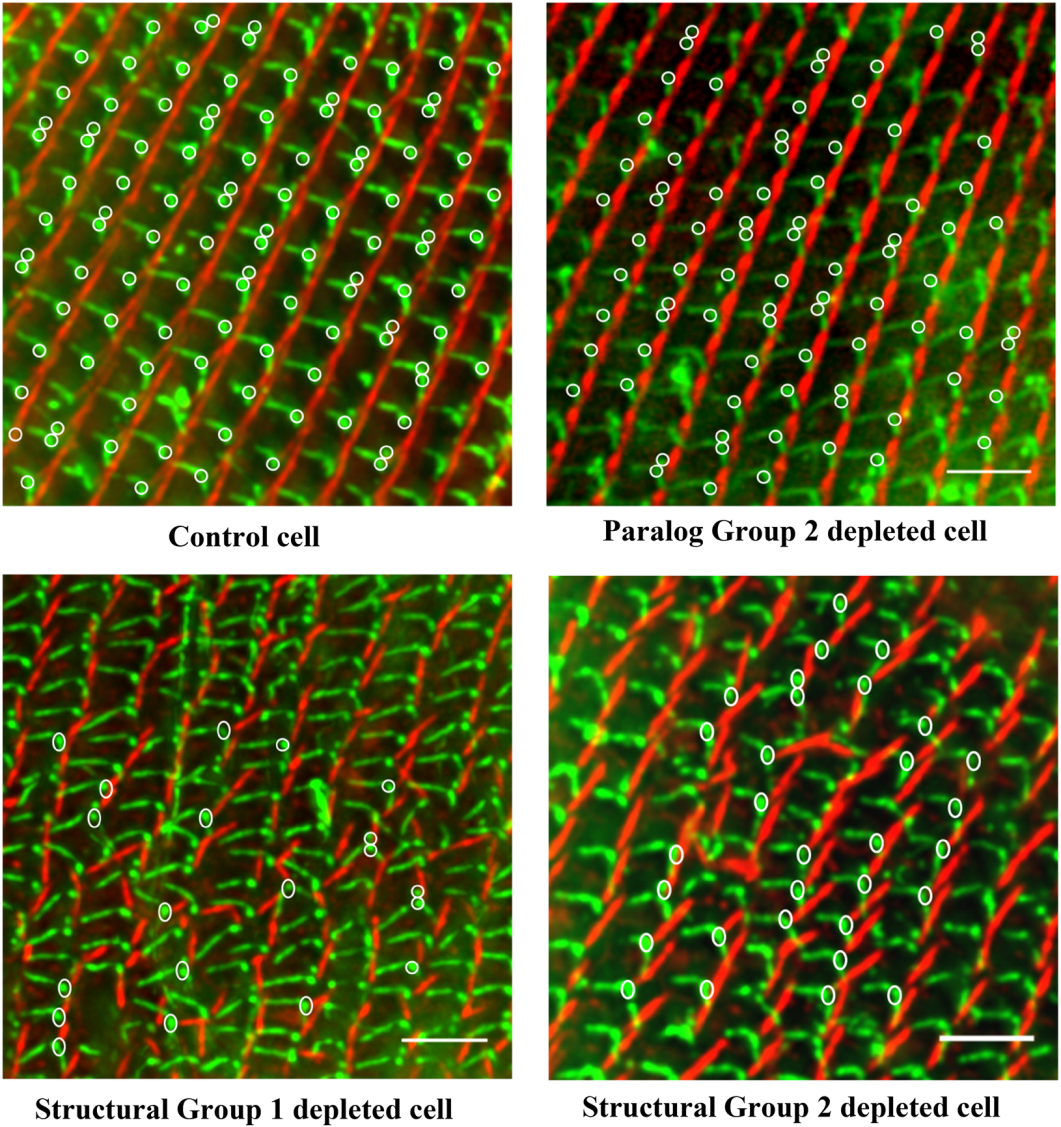
Depletion of SFA proteins leaving the angles between rootlets unaffected. Images show the staining of all three rootlets [TR (green), PR (green) and SR (red)] along with basal bodies (green) in the control, Paralog Group 2-depleted cell, Structural Group 1-depleted cell or Structural Group 2-depleted cell. Scale bars are 3 μm. White circles denote the position of the basal bodies with all three rootlets visible that were used in measuring the angles between rootlets. It took more images to reach 100 basal body measurements from the misaligned areas because we did not want to skew the measurements in these areas with basal bodies from normal rows

To visualize all three rootlets, we treated the cells with anti-α tubulin for the microtubule-based rootlets (TRs and PRs), ID5 for basal bodies and anti-SR for the SRs. Fields of basal bodies at the cell surfaces of several cells are shown in Fig. 8.

**Fig. 9:**
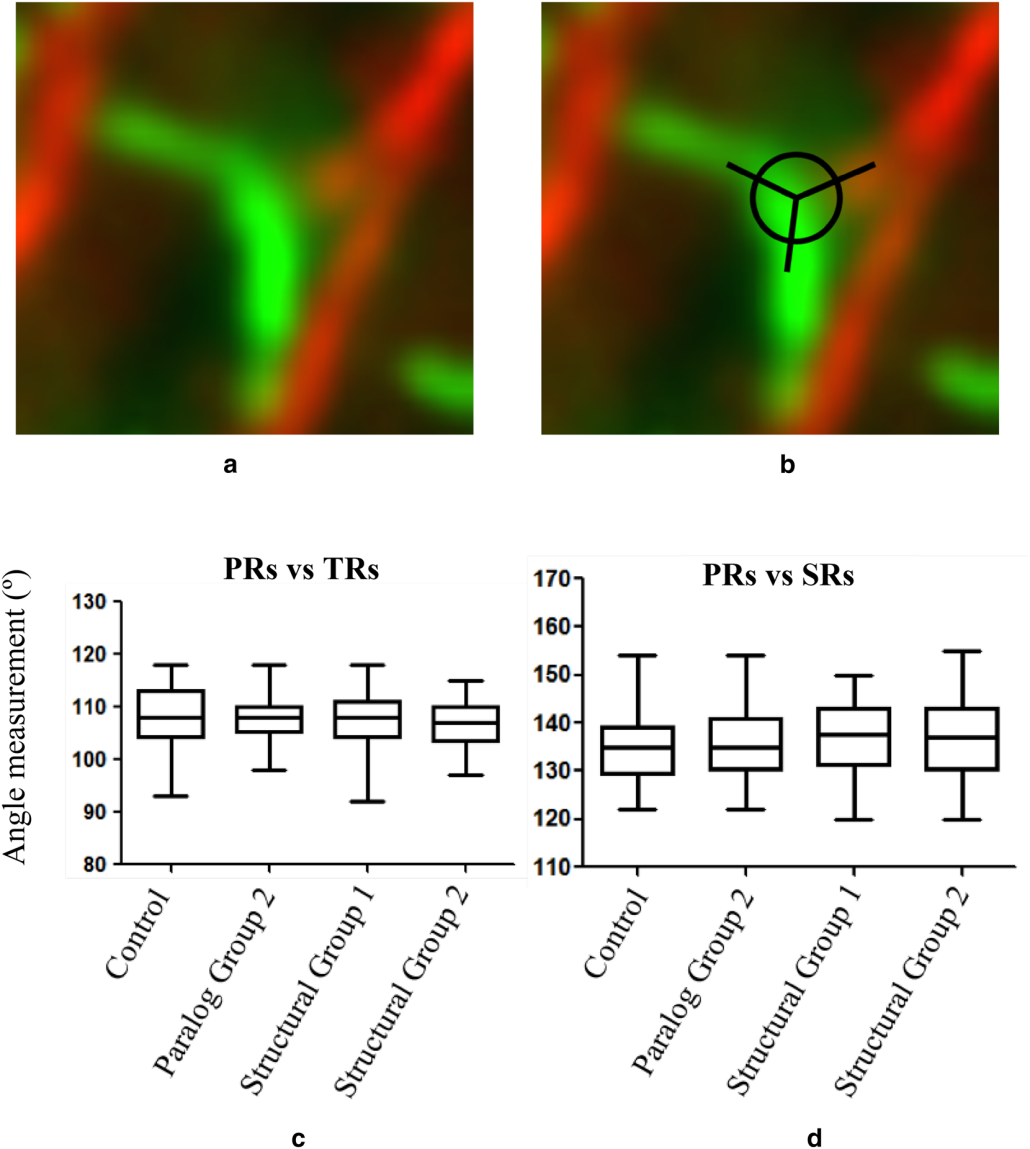
Rootlet angles relatively constant in the control and SFA protein-depleted cells. **a** The enlarged image of an individual basal body with all three rootlets [TR (green), PR (green), and SR (red)] along with basal bodies (green). **b** The method showing how the angles were measured: First a circle was drawn around the basal body; then three straight lines were drawn to determine the positions of rootlets to the basal body; finally, angles were measured by protractor. **c**, **d** The measurement of angles between PRs and TRs (different group: Mean ± SEM (standard error of the mean); Control: 108 ± 0.56; Paralog Group 2: 107.7 ± 0.44; Structural Group 1: 107.1 ± 0.43; Structural Group 107.2 ± 0.50) and between PRs and SRs (different group: Mean ± SEM; Control: 134.9 ± 0.68; Paralog Group 2: 135.9 ± 0.69; Structural Group 1: 136.7 ± 0.83; Structural Group 136.4 ± 0.77) in control, Paralog Group 2-, S-tructural Group 1-. and Structural Group 2-depleted cells, respectively. Angle values (both for PRs vs TRs and PRs vs SRs) from different groups are not statistically significant (*P* value < 0.5; unpaired *t*-test) compared to control cell

We examined angles for basal bodies from control cells and cells with depletions of a Paralog Group or a Structural Group (Structural Group 1 or 2). To analyze the angles between rootlets, we identified in Fig. 8 basal bodies that had all three rootlets visible and marked them with white circles. We selected basal bodies only in the affected surfaces area of the cells for Structural Group-depleted cells to avoid biasing the results toward control values. The angles between PRs and the TRs and between PRs and SRs showed no significant differences among basal bodies of the control cells, cells depleted of a Paralog Group or depleted of a Structural Group (Fig. 9c, d). We counted 100 basal bodies for each of the cell types, although we required more fields of basal bodies for the Structural Group-depleted images.

While examining these fields of basal bodies, we used several focal planes. Basal bodies were all in the same planes beneath the cell surface, and we observed no intracellular undocked basal bodies. Therefore, the basal bodies seemed to be docked even though misaligned.

### Phenotype 5: Mass Spectrometry correlates RNAi with depleted proteins

**Fig. 10:**
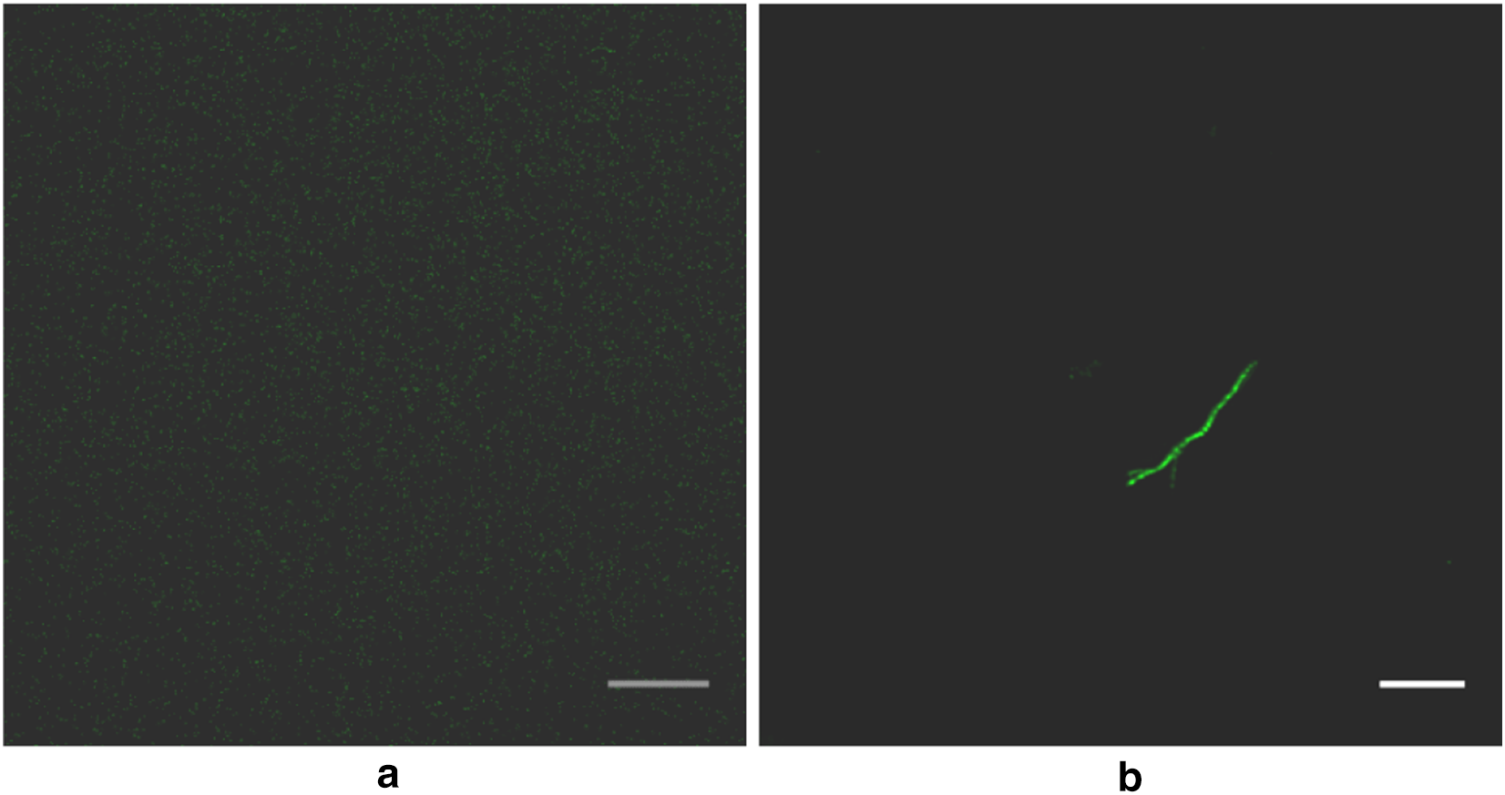
The presence of SR-type structures in the 30% Optiprep faction of cells expressing FLAG-SFA8a. The cells homogenating from FLAG-SFA8a-expressing cells prepared as described in “Materials and methods” are separated by density on an Optiprep step gradient. **a** No immunofluorescent structure in the 20% Optiprep fraction embedded in agarose. **b** A representative immunofluorescence image of what appears to be a FLAG-SFA8a-labeled SR from the 30% Optiprep fraction embedded in agarose. Scale bar is 2 μm

We used Optiprep density gradients and cell fractionation described in “Materials and methods”. To look for SR structures, we embedded Optiprep fractions from *FLAG*-*SFA8a* expressing cells in low melting agarose and used anti-FLAG antibody to visualize structures. Only the 30% Optiprep fraction had immunofluorescent structures. Figure 10a shows an image from the 20% Optiprep fraction, representative of all of the fractions except 30%. The fluorescent structures from the 30% Optiprep fraction have the size (~ 5 µm) and shape of SRs (Fig. 10b). We obtained the same results using cells expressing *FLAG*-*SFA7a* and *FLAG*-*SFA2.*

LC–MS/MS analysis following PAGE separation of the proteins from the 30% Optiprep fraction confirms the presence of SFA proteins (Additional file 2: Table S4) in the gel. [No other Optiprep fraction that we tested by LC–MS/MS identified SFA proteins (data not shown).] Using data pooled from three experiments, we identified unique peptides for SFA proteins from all Structural Groups. For example, 8 and 13 peptides were found for proteins SFA1a and 1b, respectively, which compose Structural Group 1; 13 peptides were found for SFA7a/7b, which have almost identical gene sequences and compose Structural Group 2. For other Structural Groups, we found 35 peptides for Structural Group 3, 47 peptides for Structural Group 4 and 60 peptides for Group 5. (See Additional file 2: Table S4 for more detail.)

All Paralog Groups except Paralog Group 12 had unique peptides that could be assigned to these *SFA* genes. Paralog Group 12 is in Structural Group 4, which had 47 peptides, only 12 of which could be assigned to genes uniquely. Therefore, it is possible that Paralog Group 12 members are expressed without finding unique peptides because the peptides found are also in other group members. We believe that genes from Paralog Group 12 are expressed because the cDNA needed for the RT-PCR to confirm effectiveness of RNAi for *SFA12b*, for example, is produced from converting mRNA into cDNA. Also, expressed sequence tags (ESTs) for these SFA genes were found in the ParameciumDB for expression in vegetative cells.

In other experiments, we depleted the transcripts for Structural Group 1, and analyzed the 30% Optiprep fraction by LC–MS/MS. As expected, in all three replicate experiments, mass spectrometry failed to identify peptides for the Structural Group 1, but peptides from all other Structural Groups were present. (See Additional file 2: Table S5 for more detail.) In three experiments in which we depleted Structural Group 2, we observed similar results, i.e., no proteins from the depleted Structural Group but peptides from all other Structural Groups were present. (See Additional file 2: Table S6 for more detail.)

We found non-SFA proteins in the 30% Optiprep fraction as well. α-tubulin (4 unique peptides) and β-tubulin (16 unique peptides) were found and probably signify that occasionally the basal bodies remain associated with the SRs [17]. Also found were centrin family proteins (6 peptides for the ICL1e family with 1 peptide unique to Ptcen12) and centrin binding proteins (21 unique peptides for PrCenBP1). The presence of centrin was of interest since centrin is a major protein in one of the two types of rootlets *Chlamydomonas* [22]. In addition, centrin deficiency in *Chlamydomonas* causes defects in the flagellar root system [35]. Noteworthy is that we did not find any SRL proteins in our LC–MS/MS analysis of these fractions.

It is important to note that we are not presenting the Optiprep density fractionation as a purification of the SR structures. There may be additional structural proteins of the SR beside the SFA proteins, which we have shown by epitope tagging to be in the SRs (Fig. 3). Also, there may be cortex proteins in this Optiprep fraction unrelated to the SR. Nonetheless, the structures that we purport to be SRs have the dimensions and striations (below) expected of an SR established by others [18, 36]. Also noteworthy is that we never found peptides from any SR-like (SRL) proteins through the mass spectrometry analysis of the Optiprep fractions.

### Phenotype 6: Cells depleted of Structural Groups show abnormal SR length and striations

We used negative staining and transmission electron microscopy (TEM) to examine whole SRs from the Optiprep preparation from control, Paralog Group-, or Structural Group-deplete d cells. We examined 20 or more structures for each experiment, which were repeated three times, allowing us to evaluate at least 60 structures per experimental condition. (The images with scale bars were provided by the Delaware Biotechnology Institute.)

**Fig. 11:**
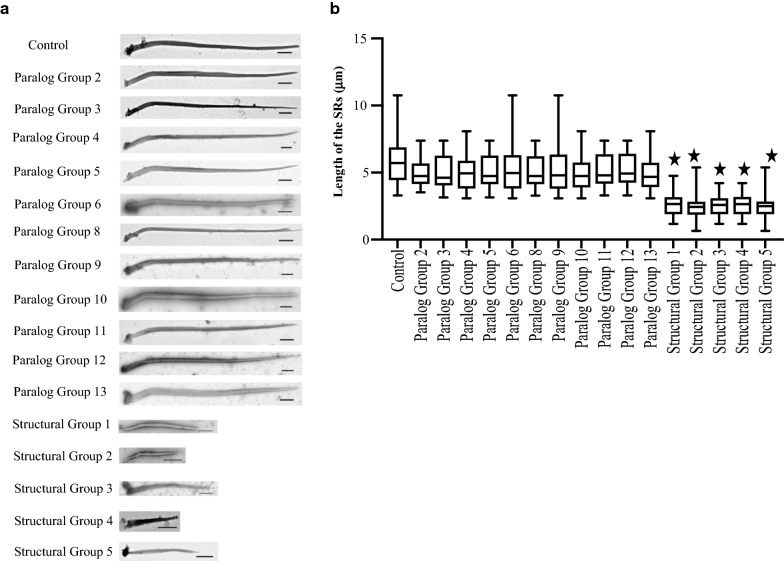
Depletion of Structural Groups causing changes in the length of the SRs. **a** Transmission electron micrographs of the negatively stained SRs from Control, Paralog Group-, or Structural Group-depleted cells. All panels show representative images from 60 SRs (20 per experiment, each done in triplicate). **a** the representative images of SRs from Control cells, those depleted of Paralog Groups 2–13, and those cells depleted of Structural Groups 1–5. The original images in Structural Groups 1–5 were reduced to match the scale bars for the images from Paralog Group-depleted cells. Scale bars are 0.5 μm. **b** The length comparison of isolated SRs. In the graph, each bar indicates the mean length with standard deviation of the SRs isolated from different groups. Asterisks indicate lengths of SRs from Structural Groups (1–5)-depleted cells are significantly (*P* value < 0.005; unpaired *t*-test) shorter than lengths from Control or Paralog Groups

The structures that we purport to be SRs from L4440-fed control cells are long (Fig. 11a). Figure 11b shows the average length measurements of control cell SRs as 5.5 µm ± 1.8 µm S.D. The range of lengths is from 3.5 to 11 µm. These values overlap the length range given by Sperling of 8–10 µm [17, 18]. The bends in the structures occur approximately where Hufnagel describes, i.e., SRs are slightly bent fibers in which tapering and a bend start at approximately 700 nm (Fig. 11a). Hufnagel describes the variability especially of the SR lengths and widths depending upon the area of the cortex in which they are found. Sperling describes the dynamic nature of the SRs that can lead to differences in length estimates [18]. Regardless of the variations in dimensions, the hallmark of the SR is the pattern of striations as reviewed by Hufnagel [36]. Therefore, we refer to the striated structures below as SRs.

In the cells depleted of Paralog Groups that are not also Structural Groups (Paralog Groups 2 through 6, and 8 through 13), the lengths of SRs are close to that of control cells (Fig. 11a). Strikingly, the depletion of individual Structural Groups (1, 2, 3, 4, or 5) correlates with the shortening of the SRs (Fig. 11b). The graph in Fig. 11 shows that there is some variability in length for each condition; the individual SRs that were chosen as representative were within each size range, but we did not select them to be at mid-range.

Note also that the SRs in Fig. 11a Structural Groups 1–5 show unusual shapes, possibly consistent with the unusual forms in Figs. 4d, 5a, m–o and 6e, f.

We caution that while the IF images such as Fig. 4d seem to show shortened SRs, it is difficult and perhaps impossible to obtain accurate length measurements from these images. The control image in Fig. 4b has what look like SRs truncated and not in the overlapping cable as in Fig. 5c. This distortion is due in part to the curvature of the cell surface and focal planes in the image stacks. We consider that Figs. 4d and 5m–o probably shows shorter SR, but it would be difficult to obtain good quantitative data from these images. Therefore, we favor using the TEM data for length measurements.

**Fig. 12:**
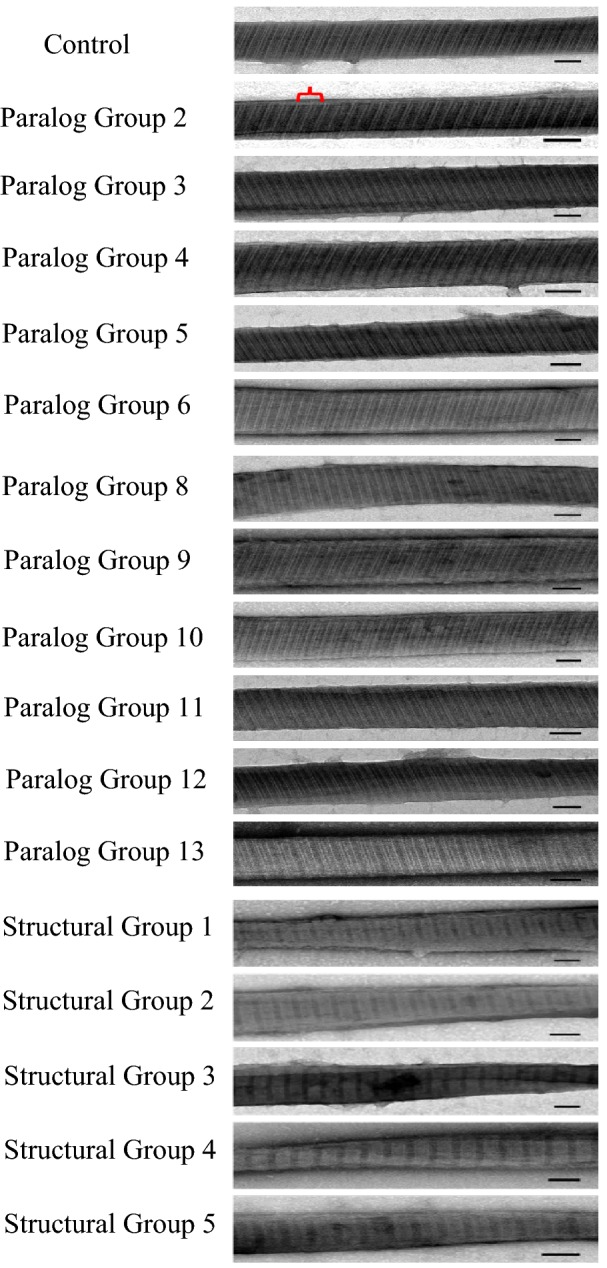
Depletion of Structural Groups causing changes in the striation pattern of SRs. Panels show representative transmission electron microscopy images of the striation pattern of negatively stained SRs from Control, Paralog Group- or Structural Group-depleted cells. In Control and Paralog Group-depleted cells, major striations occur at 24–36 nm intervals (red bracket). Scale bars are 50 nm. In Structural Group-depleted cells, the striation pattern appears altered

We also used TEM to examine the striation patterns of SRs. In control cells or cells depleted of Paralog Groups, the striation pattern of their SRs showed typical periodicity in which the major striation occurs at 24–36 nm intervals (Fig. 12, indicated by red bracket) closely matching that seen by Hufnagel [36]. In SRs from cells depleted of any Structural Group, the striation patterns changed significantly. Regardless of the Structural Group depletion, 40–60% of the structures examined had some striation bands missing from the repeating unit (Fig. 12), and, in the remainder of the structures identified by TEM, striations were lost altogether (data not shown). That is, none of the SRs had a normal striation pattern. However, we provide images of only structures with changed striation patterns and not the ones devoid of all striations because we realize that there could be skepticism about whether these latter structures are valid SRs. We did not see structures like those without striations in the control preparations. Also, the structures without striations often look frayed.

## Discussion

The mammalian SR is a one of the largest cytoskeleton structures, originating from the base of the cilium and extending toward the cell nucleus [11, 37]. Rootletin, a 220 kDa protein with an N-terminal globular head domain and C-terminal coiled-coil domain is a structural component of the mammalian rootlet [37]. Ciliated cells in mice with targeted disruption of the rootletin gene are devoid of rootlets [11]. The main function of the SR in the mammalian system is to provide structural stability for the cilium [11, 37].

*Chlamydomonas* has two types of flagellar roots, one with SF-Assemblin as the major protein, the other with centrin [21, 22]. As with Rootletin’s structure, the coiled-coil domain is a hallmark of *Chlamydomonas* SF-Assemblin. Therefore, we also focused on the coiled-coil domains in *Paramecium SF*-*Assemblin* homologs and found that the secondary and tertiary structures of the putative *SFA* genes were organizing principles for their function into what we call Structural Groups.

The *Paramecium* SR emanates from the left side of the proximal end of the basal body and stretches upward toward the surface [14, 20]. The SRs follow a line from the basal body toward the anterior of the cell, through the cortical unit ridge and past several more basal bodies and cortical units. As shown in [14], two or three SRs can be found in parallel in the ridge on the left side of the cortical unit but not touching each other as they course toward the anterior. These large structures have been shown to be striated, dynamic and change length during the cell cycle [17, 18, 36].

Using the *SF*-*Assemblin* gene [21] we found 30 *Paramecium* genes that appear to code for SFA proteins. The large number of genes reflects the three whole genome duplications in *Paramecium* [38]. As we show here, *SFA* genes can be assigned to 13 Paralog Groups that can be combined into five Structural Groups. All members of a single Paralog Group belong to the same Structural Group (Table 1, Fig. 2). Structural Group members are related by nucleic acid and amino acid sequences and the potential for coiled-coil domains in the same part of their proteins.

It is important to note that we have focused on genes with homology to SF-Assemblin, and have not shown that the SR is composed of only these proteins. Nonetheless, we selected one gene from each of the 13 Paralog Groups for epitope tagging and showed that the tagged expressed proteins are found in the physical SR. In contrast, tagged and expressed SRL proteins that lack the homologous SF-Assemblin sequences, are found elsewhere in the cell, including in cytoplasm, epiplasm and cilia (Additional file 1: Fig. S3).

In light of the large number of SFA proteins and their interesting sequence organization, our motivation became to identify the relationships among the many *SFA* genes and the multiple phenotypes of RNAi depletions of the groupings of genes into Paralog and Structural Groups (Fig. 2). RNAi silencing of multiple genes from these groups presented challenges to precisely target only the gene(s) of interest. We used RT-PCR to assiduously check on our accuracy. The results of these RNAi depletions led to the descriptions of the following phenotypes:

Phenotype 1: Cells depleted of Structural Groups show basal body row misalignment, SR row misalignment, and abnormal gross SR appearance. These misalignments can be seen anywhere on the cell surface except the oral groove. Interestingly, these misalignments correlate with the depletion of any Structural Group, as we discuss further below.

Phenotype 2: Cells depleted of Structural Groups show distorted cortical units. The rows of cortical units track with the rows of basal bodies and it might be expected that they both become misaligned together. However, the cortical units also show distortion of their normal rectangular shape beyond misalignments.

Phenotype 3: Cells depleted of structural groups are ciliated and able to swim. Even though the basal body rows and cortical units are not properly aligned, the cells are ciliated. Even in areas of severe misalignment, cilia can be seen with immunofluorescence to emanate from basal bodies. These results imply that the basal bodies are docked properly at the cell surface.

Additional evidence of ciliation is that the depleted cells are able to swim, albeit with inefficient and loopy paths (Fig. 5h). Such paths are expected if the cilia no longer pull with their power stroke in metachronal waves toward the posterior, but instead pull in multiple directions working against each other. If only basal bodies that remain in their straight rows are motile, this phenotype would not occur. The cells might swim slowly, but their paths would be straight. It requires that some cilia pull against or across the normal power stroke toward the posterior. Again, this evidences that the basal bodies of the affected areas of the surface are docked at the surface membrane.

Phenotype 4: Basal bodies in cells depleted of Structural Group proteins have rootlets emerging at normal angles. The basal bodies seem to be sufficiently normal to have two microtubule rootlets and an SR attached at the proper microtubule triplets. While we cannot distinguish the specific triplets that serve as attachment sites on the basal body, we can determine whether the expected angles among them are maintained to be consistent with: PC attached to triplet 9/1; TR attached to triplet 5; and SR attached to triplet 6–7 [34]. The loss of row alignment by the basal bodies does not appear to be due to loss of rootlet attachment sites on the basal body.

In these rootlet angle studies, we observed no internal basal bodies; all seem to be docked at the membrane. Since the rootlets and basal bodies could not be visualized in the same focal planes, multiple Z sections were needed for this analysis. However, the number of sections needed to visualize the rootlets and basal bodies did not differ among the control and RNAi treated cells. We take these observations as inconsistent with failure of basal bodies to dock. In all other IF studies of whole cells, likewise, we saw no evidence of undocked or internal basal bodies.

Phenotype 5: Mass spectrometry correlates RNAi with depleted proteins. Only the Optiprep fraction in which the epitope tagged fluorescent SRs are found also harbors peptides from all groups of the SFA proteins as shown by LC–MS/MS. When RNAi-depleted cells are used for cell fractionation, the peptides from proteins targeted in the depletion are no longer found by LC–MS/MS in the 30% Optiprep fraction. Other proteins can be found in this Optiprep fraction, but we have no indication that they are integral to the SR.

Phenotype 6: Cells depleted of Structural Groups show short SR length and abnormal striations. There appears to be redundancy among the Paralog and Structural Group members in that single proteins or even whole Paralog Groups can be depleted with no statistically significant shortening or visually assessed disruption of the striations of the SR. Even with the depletion of a Structural Group of proteins, there is sufficient redundancy such that a large structure is formed, albeit with shortened length and abnormal striations.

The coiled-coil regions of the SFA proteins likely are responsible for the protein–protein interactions that link them into fibers with darker striations where the proteins overlap. It is possible to make a model that utilizes one protein from each structural group to create a fiber of repeating units that has major and minor striations in a 24–34 nm period [39]. However, this is speculative and not tested.

How reductions by RNAi in the amount of SFA transcripts and proteins cause the characteristic misalignments of basal bodies and cortical units is not yet clear. We propose that the shortened SR, caused by Structural Group RNAi, cannot properly support the basal bodies through forces from beating cilia. The basal bodies consequently rotate out of alignment, taking their rootlets with them and distorting the cortical units.

Our proposal and results fit well with the work of Pearson [13] about the role of the protein DisAp in basal body orientation in multiciliary arrays of *Tetrahymena*. The protein DisAp is not an SF-Assemblin homolog, but is associated with the *Tetrahymena* SR. Their studies show that DisAp prevents basal body rotation and also maintains the necessary SR length to protect the basal body. In the disA-1 mutant cells, the SR is shorter, making the basal bodies susceptible to rotation due to the forces put on them by beating cilia.

For *Tetrahymena*, it is proposed that the SR length is important in the contact with and anchoring to structures in the cortex to resist the ciliary forces that would rotate basal bodies out of alignment. We propose that the changes in the *Paramecium* SR length due to depletion of SFA proteins from a Structural Group changes how the basal body is secured. The beating cilia, consequently, would provide the forces to move the basal bodies out of their normal alignment. However, for *Paramecium*, there is a second aspect to consider in addition to shortening of the SR: the loss of specific proteins that is manifest in a changed striation pattern.

DisAp is not an SF-Assemblin homolog [13], and not among proteins found in our LC–MS/MS analyses. Nonetheless, our results of the RNAi for Structural Groups are compatible with those of the *Tetrahymena* SR. In both studies, shortened SRs are associated with misalignment of the SR, basal body and cortical unit rows. For *Tetrahymena*, the authors found that ciliary force is necessary and sufficient to misalign basal bodies in dis-A1 mutant cells and that normally basal body rotation out of alignment is resisted by the SR.

In our studies, it appears that the basal bodies dock at the surface and have all three rootlets emanating at normal angles in the Structural Group-depleted cells. As proposed for *Tetrahymena*, our results imply that the SR has lost an attachment that normally anchors the basal body in the correct orientation. We propose that the shortened SR cannot reach its target to make an attachment, but it is also possible that specific SR proteins or the striation components participate in such an attachment. There are many potential attachment partners to investigate, including those in epiplasm, cortical unit ridges, ICL and anterior basal body microtubule rootlets. Our discovery that the SRs can be reliably and systematically changed will facilitate these kinds of investigations.

## Conclusions

These studies have elucidated *SFA* homologous genes that code for components of the *Paramecium* SR and the functional relationships among the groups of these genes. We found that the depletion of Structural Groups led to the dramatic phenotypes of loss of basal body row orientation, cortical unit organization, and the SR shape, striations and length. We have provided the general relationships of SFA proteins from Paralog and Structural Groups that should be taken into account in models of how the many SFA proteins could interact to form filaments and a striated SR. Our study has made it possible to consistently disrupt the *Paramecium* SFAs by RNAi and characterize phenotypes of this disruption.

In our studies, it appears that the basal bodies dock at the surface and have all three rootlets emanating at normal angles in the Structural Group-depleted cells. These results imply that the SR has lost an attachment to a cell surface or cortex component that normally anchors it and resists the forces from beating cilia. Beyond proposing that the length of the SR is critical in making the proper attachment, we do not know which of the SFA proteins or the striation components might participate in the attachment. There are many potential attachment partners to investigate. To facilitate the future identification of these components of structures such as those from ICLs, epiplasm or cortical unit ridges, our discovery of the phenotypes of RNAi for Structural Groups provides a systematic way to manipulate the SRs.

## Supplementary information

**Additional file 1: Fig. S1.** An example of the RT-PCR analysis showing the depletion of mRNA of a targeted Structural Group. **Fig. S2.** The phylogenetic relationships among the *SFA* and *SRL* genes in *P. tetraurelia*. **Fig. S3.** SRL proteins have locations that differ from those of SFAs (Fig. 3).

**Additional file 2: Table S1** RNAi constructs and nucleotides. **Table S2.** Primers used in the study. **Table S3.** Predicted Positions of the coiled-coil domains in the SFA proteins and Accession Numbers. **Table S4.** SFA proteins found by LC–MS/MS of an Optiprep density faction from Control Cells (combined from 3 experiments). **Table S5.** SFA proteins found by LC–MS/MS, analysis of an Optiprep density faction from Structural Group 1 depleted cell (data combined from 3 experiments). **Table S6.** SFA proteins found by LC-MS/MS found by LC–MS/MS analysis of an Optiprep density faction from Structural Group 2 depleted cell (data combined from 3 experiments).

## Data Availability

All data generated or analyzed during this study are included in this published article (and its additional information files). The datasets used and/or analyzed (LC–MS/MS, and rootlet angle counting data) during the current study are available from the corresponding author on reasonable request.

## Abbreviations

SR: striated rootlet
PR: postciliary rootlet
TR: transverse rootlet
SFA: SF-Assemblin homologs
MS: mass spectrometry
