# Supplementary material for: SF-Assemblin genes in Paramecium: phylogeny and phenotypes of RNAi silencing on the ciliary-striated rootlets and surface organization
Source: Cilia. 2019 Oct 29;8:2. doi: 10.1186/s13630-019-0062-y (PMC6819543; doi:10.1186/s13630-019-0062-y)
Supplement: Supplementary file 1 — Additional file 1: Fig. S1. An example of the RT-PCR analysis showing the depletion of mRNA of a targeted Structural Group. Fig. S2. The phylogenetic relationships among the SFA and SRL genes in P. tetraurelia. Fig. S3. SRL proteins have locations that differ from those of SFAs (Fig. 3). [file 13630_2019_62_MOESM1_ESM.docx]

Additional File 1: Fig. S1 An example of the RT-PCR analysis showing the depletion of mRNA of a targeted Structural Group.

Representative images of agarose gels demonstrating the level of mRNA depletion using gene-specific primers and RT-PCR. In this example, we are targeting the two genes in Structural Group 1 (*SFA1a* and *SFA1b*). For each panel, the left side shows the cDNA in a dilution series after RNAi using control bacteria; the right side shows the cDNA in a dilution series after RNAi for Structural Group 1 genes. The Panel A (top) shows the effects on cDNA for a control gene, calmodulin. Note that there is no apparent difference in the intensity of the bands at the various dilutions between the control (left) and test (right) cDNA, suggesting that the cDNA of the two samples (test and control) are of approximately equal concentrations. Panels B and C show that there is a clear reduction in the band intensity of the serial diluted cDNA following RNAi for the Structural Groups (right) when compared to the control cDNA (left) using gene-specific primers (*SF1a* and *SFA1b*). Panel D shows that the amplification using primers for an unrelated *SFA* gene (*SFA7a)* shows no difference between cDNA from the Control or the Structural Group 1 RNAi treatment.

cDNA was diluted for the template at 1:10 and 1:100 in lanes a and b respectively, and at 1:500 and 1:1000 in lanes c and d respectively (with the exception of Panel A for calmodulin that omits the 1:1000 dilution).

In addition, a control 1:10 dilution of cDNA where the reverse transcriptase was omitted after extraction of mRNA is found in Panels A (lane d) and Panels B-D, lane e.

In all panels, m indicates the 100bp DNA Ladder. RT-PCR was repeated three times for every RNAi experiment, checking on all of the messages in the Structural Group and additional controls for off-target effects.


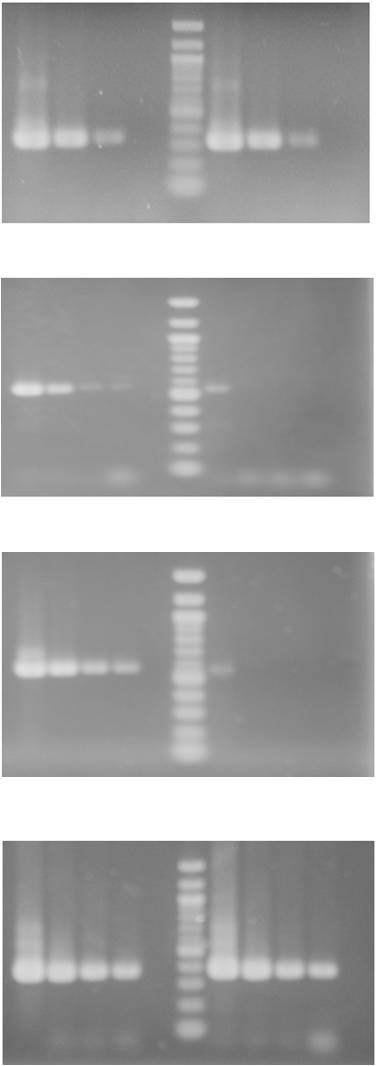


Control

RNAi Structural Group depleted cells

a b c d m a b c d

a b c d e m a b c d e

a b c d e m a b c d e

a b c d e m a b c d e

cDNA dilutions

cDNA dilutions

cDNA dilutions

cDNA dilutions

A

*Calmodulin* gene

B

*SFA1a* gene

C

*SFA1b* gene

D

*SFA7a* gene

Additional File 1: Fig. S2 The phylogenetic relationships among the *SFA* and *SRL* genes in *P. tetraurelia*.

Analysis of the 30 *SFA* genes and 11 *SRL* genes was conducted in MEGA6 (Tamura et al., 2013). The evolutionary history was inferred using the Neighbor-Joining method (Saitun and Nei, 1987)­­. The analysis involved all 41 nucleotide sequences. The final dataset includes 475 positions and eliminates all positions having the gap and missing data sets.


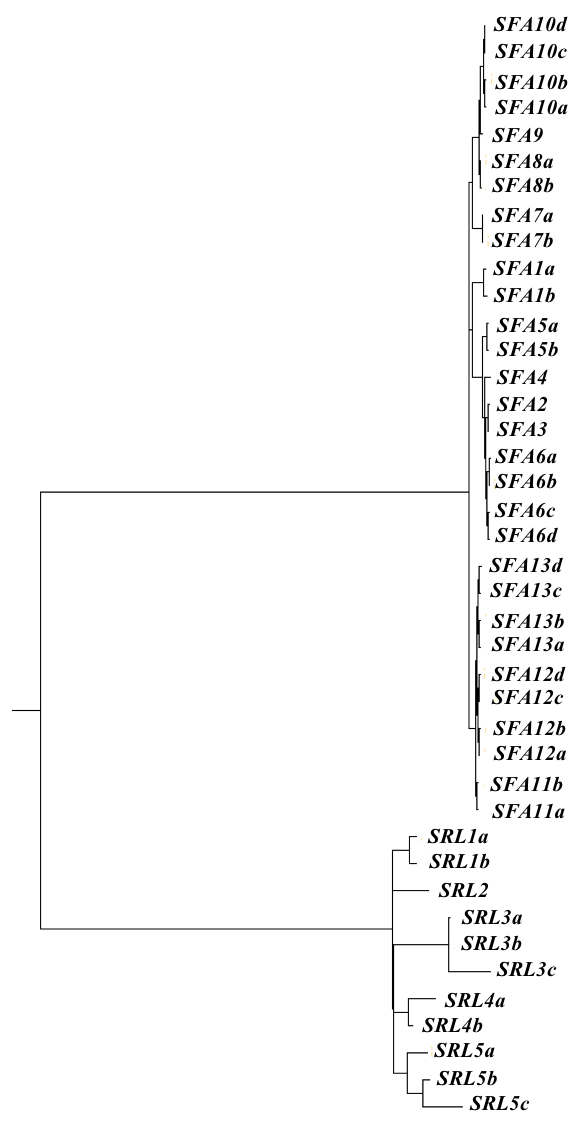


Additional File 1: Fig. S3 SRL proteins have locations that differ from those of SFAs (Fig. 3).

Panel A shows the whole cell image of cells each expressing one of four FLAG-SRL proteins (green) that are representative of the 4 Paralog Groups of SRLs. FLAG-SRLs are indicated in green; SRs in the top two panels and basal bodies in the bottom two panels are in red. SRs are visualized with anti-SR antibody (red).

In Panel A, the FLAG-SRL1a is located intracellularly whereas FLAG-SRL3a is located in the cilia. Both FLAG-SRL4a and FLAG-SRL5a are located in the epiplasm of the *Paramecium* cell. Scale bars are 15μm.

Panel B shows the side view images of FLAG-SRL expressing cells. FLAG-SRLs are in green; SRs in the top two panels are in red (anti-SR antibody). In the bottom two images, basal bodies are in red in the bottom two panels (anti-basal body centrin antibody). FLAG-SRL1a is located intracellularly. FLAG-SRL3a appears to be punctate in the cilia and basal bodies. FLAG-SRL4a and 5a are located in the epiplasm.

Scale bars are 3μm.

SRL4a


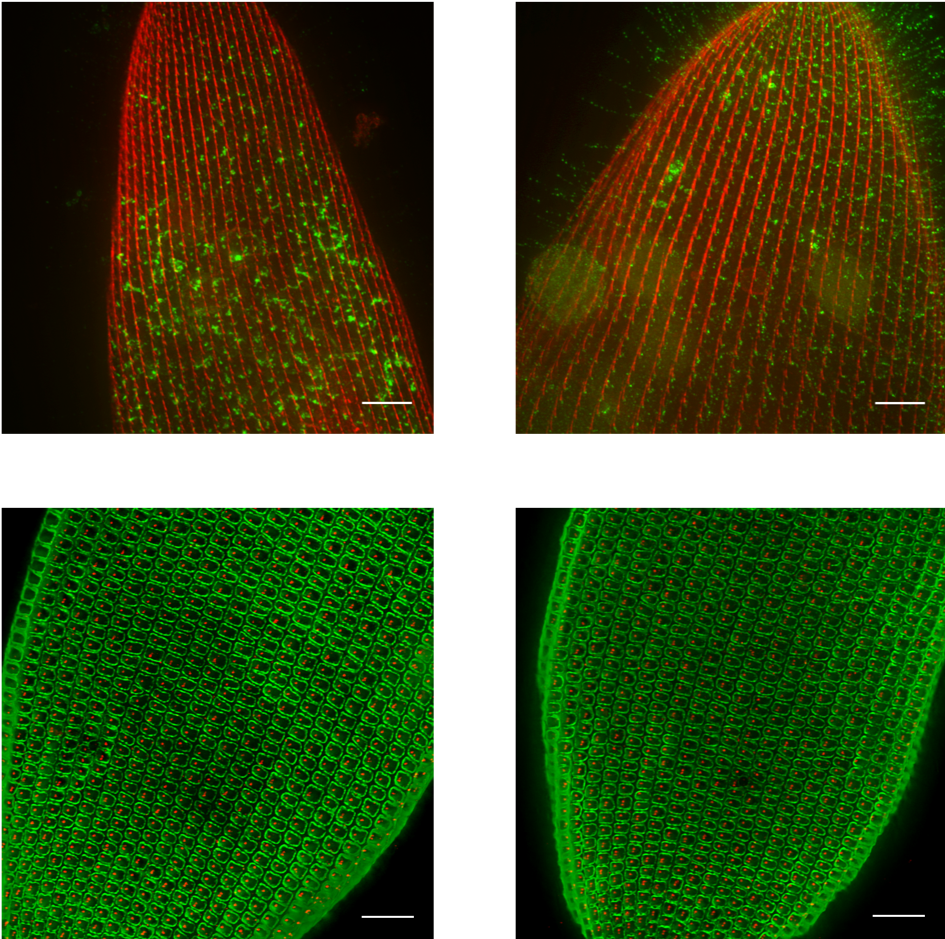


SRL1a

SRL3a

SRL5a

A

SRL1a

SRL5a

SRL3a

SRL4a


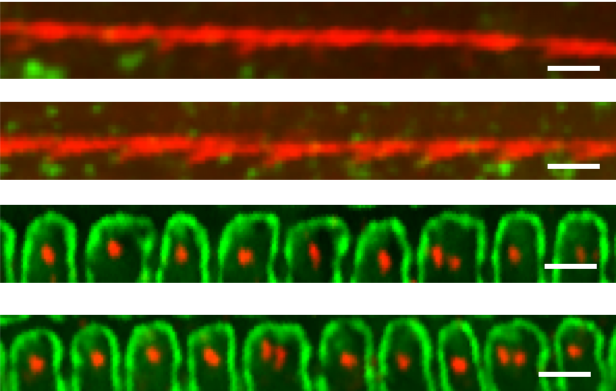


B
