# Supplementary material for: SF-Assemblin genes in Paramecium: phylogeny and phenotypes of RNAi silencing on the ciliary-striated rootlets and surface organization
Source: Cilia. 2019 Oct 29;8:2. doi: 10.1186/s13630-019-0062-y (PMC6819543; doi:10.1186/s13630-019-0062-y)
Supplement: Supplementary file 2 — Additional file 2: Table S1 RNAi constructs and nucleotides. Table S2. Primers used in the study. Table S3. Predicted Positions of the coiled-coil domains in the SFA proteins and Accession Numbers. Table S4. SFA proteins found by LC–MS/MS of an Optiprep density faction from Control Cells (combined from 3 experiments). Table S5. SFA proteins found by LC–MS/MS, analysis of an Optiprep density faction from Structural Group 1 depleted cell (data combined from 3 experiments). Table S6. SFA proteins found by LC-MS/MS found by LC–MS/MS analysis of an Optiprep density faction from Structural Group 2 depleted cell (data combined from 3 experiments). [file 13630_2019_62_MOESM2_ESM.docx]

Additional File 2: Supplemental Table 1 RNAi constructs and nucleotides.

| Name | Accession Number | Nucleotide base position in gene sequence |
| --- | --- | --- |
| *SFA1a* | GSPATG00025723001 | +253 - +842 |
| *SFA1b* | GSPATG00023935001 | +257 - +870 |
| *SFA2* | GSPATG00020957001 | +23 - +790 |
| *SFA3* | GSPATG00022867001 | +163 - +786 |
| *SFA4* | GSPATG00008626001 | +3 - +805 |
| *SFA5a* | GSPATG00026454001 | +27 - +632 |
| *SFA5b* | GSPATG00024123001 | +7 - +716 |
| *SFA6a* | GSPATG00010780001 | +341 - +800 |
| *SFA6b* | GSPATG00014274001 | +114 - +726 |
| *SFA6d* | GSPATG00014806001 | +174 - +632 |
| *SFA7a* | GSPATG00036966001 | +178 - +680 |
| *SFA8a* | GSPATT00032447001 | +14 - +864 |
| *SFA8b* | GSPATG00033989001 | +64 - +740 |
| *SFA9* | GSPATT00011977001 | +59 - +862 |
| *SFA10a* | GSPATG00034929001 | +113 - +439 |
| *SFA10d* | PTETG17800004001 | +364- +820 |
| *SFA11a* | GSPATG00002032001 | +39 - +690 |
| *SFA11b* | GSPATG00017545001 | +115 - +698 |
| *SFA12a* | GSPATG00001334001 | +132 - +793 |
| *SFA12b* | GSPATG00002707001 | +87 - +791 |
| *SFA12d* | GSPATG00004838001 | +141 - +728 |
| *SFA13a* | GSPATG00025686001 | +127 - +799 |
| *SFA13c* | GSPATG00020721001 | +122 - +712 |
| *SFA13d* | GSPATG00021965001 | +116 - +694 |

Additional File 2: Supplemental Table 2 Primers used in the study.

| Name |  | Primers  5’to 3’ |
| --- | --- | --- |
| *SFA1a* | Forward | GGCAGACGACTCAAAATTTAAG |
|  | Reverse | GACTCTCTTCAGTTGCTTCTC |
| *SFA1b* | Forward | GGAGATGATTCTAAGTTCAAG |
|  | Reverse | CATGTTTGATCGATCAAATTG |
| *SFA2* | Forward | GAATAAGAACAGGAGAAG |
|  | Reverse | CTTCTTGCATGCCTTTCC |
| *SFA3* | Forward | CATTAGTCAGATCAGAAGG |
|  | Reverse | CACTAATGTTTCCTCTGTC |
| *SFA4* | Forward | GTCTGAATCCAATGTTAAAG |
|  | Reverse | GTACAAGAGTCTCTTCTGTC |
| *SFA5a* | Forward | GACAATGTATAAACTAGAA |
|  | Reverse | CAAATAATTCTCTTAGGTCTG |
| *SFA5b* | Forward | GACAATCTATAAACTAGAGTA AC |
|  | Reverse | CAGTAATTCTAAATTACACTTCTC |
| *SFA6a* | Forward | GATTGATCGAATAGAATTCC |
|  | Reverse | CGACTAATTAGACTAAGG |
| *SFA6b* | Forward | CGATTCCATGCTATCAGCC |
|  | Reverse | GATTTCTCTCAGACTTTCCAC |
| *SFA6c* | Forward | CATCTCTACTAGTGTTTAGG |
|  | Reverse | CCTTCTCAACCAACTGCAC |
| *SFA6d* | Forward | GAGGCTTAATTGTAACATC |
|  | Reverse | CGACAAATTAATGGTATATC |
| *SFA7a* | Forward | GAGGAATCAGAGACAAGATTAG |
|  | Reverse | GTCTTTATAGCTTCATGTAATC |
| *SFA8a* | Forward | GATCCTAAGTCTCATGTATC |
|  | Reverse | CTGCAATACTGAGTTTATTAC |
| *SFA8b* | Forward | GCATCACCATCAAGTGGAAG |
|  | Reverse | CTCTATTGACGATATCTTTAAGC |
| *SFA9* | Forward | GTCTCCAAGCAGTGGAAG |
|  | Reverse | CTCTGTTTCTCTTCTCCAC |
| *SFA10a* | Forward | CCAGATAGGAGTGGAGAATG |
|  | Reverse | CCTCTGTCAATCTAAGAATC |
| *SFA10b* | Forward | GATCTCAGAGAAGCTGTCAAC |
|  | Reverse | CTGGTTCTCTTCTCTTGATCC |
| *SFA10c* | Forward | CTGCAGTGTAATATTGTGAGTG |
|  | Reverse | CAGATTGCTCTGTGGTTCTC |
| *SFA10d* | Forward | GACCAAGAAGTTCGAGAATGC |
|  | Reverse | CTAGTGTAATGAAGATGGATACC |
| *SFA11a* | Forward | CAATCCAACTACATCACGATAG |
|  | Reverse | GCATCTCAAATATTGCTGATTCAC |
| *SFA11b* | Forward | CGTGTTTCTGGTGTAGAAGAG |
|  | Reverse | CCTTGAGCATCTCAAATATTGC |
| *SFA12a* | Forward | GCGGCTAGCGGAGAGAATTTAAGACAC |
|  | Reverse | GCGGGTACCCAAGCATCTTCCAATAAT |
| *SFA12b* | Forward | GGCACACAGAATAGATACGG |
|  | Reverse | CATGCATCTTCTAACAGTCC |
| *SFA12c* | Forward | GAGAGAATGCAAGACACG |
|  | Reverse | CAAGCATCT TCTAGAAGTCC |
| *SFA12d* | Forward | GCAAGACACAATTACCTC |
|  | Reverse | CGATTTCGCTCTTGACTC |
| *SFA13a* | Forward | GTAGAGGAGAGAACTCAAG |
|  | Reverse | CAGCATCTTCGAGTAATCC |
| *SFA13c* | Forward | GCAGTAGAGGAGAGAATTCAG |
|  | Reverse | CAAAGATAGCCGTCTCGG |
| *SFA13b* | Forward | CAGCAGTTGAAGAGAGAAC |
|  | Reverse | CTAGATCCTTGAGCATCTC |
| *SFA13d* | Forward | GAGTCACAGCAGTAGAAGAGAG |
|  | Reverse | CATTTCAAAGATTGCTGTCTCC |
| *FLAG-SFA1a* | Forward | CGCGGGCCCATGTCTGCATCTAAAATC |
|  | Reverse | CGCGAGCTC TCATTTTCTTAATGAATTTTC |
| *FLAG-SFA2* | Forward | GCGGCTAGCATGTCTGAGAATATCTAAAATAGAG |
|  | Reverse | GCGGGTACCTCATAAATTCATTTCTAACATTTCTC |
| *FLAG-SFA3* | Forward | GCGGCTAGCATGTCTGAAAGTATCTAAAATAG |
|  | Reverse | GCGGGTACCTCATAAATTCATTTCTAACATTTC |
| *FLAG-SFA4* | Forward | CGCGGGCCCATGTCTGAATCCAATGTTA |
|  | Reverse | CGCGAGCTCTCATAAATTCATTTCTAAC |
| *FLAG-SFA5a* | Forward | CGCGGGCCCATGAATGACAATCTATAAAC |
|  | Reverse | CGCGAGCTCTCACATATTCATTTCTAAC |
| *FLAG-SFA6b* | Forward | GCGGGTACCTCATAAGTTCATTTCTAACATTTC |
|  | Reverse | GCGGAGCTCATGTCAGAAAATATCTAAAATAGAG |
| *FLAG-SFA7a* | Forward | GCGGCTAGCATGTCAAATAATAGAAG |
|  | Reverse | GCGGGTACCTCATGCTAGTTAAGC |
| FLAG-SFA8a | Forward | GCGGCTAGCATGTACAATTCAAGATCCTAAG |
|  | Reverse | GCGGGTACCTCATAAATTTGCTGCAATACTG |
| *FLAG-SFA9* | Forward | GCGGCTAGCATGTATAATTCAAGATC |
|  | Reverse | GCGGGTACCTCATAGATTTGCTGC |
| *FLAG-SFA10a* | Forward | GCGGCTAGCATGTCCAGAGTCAAACAATC |
|  | Reverse | GCGGGTACCTCATAAATTTGCTGCAATGC |
| *FLAG-SFA11a* | Forward | GCGGCTAGCATGAATCCAAATACACCATTCAACG |
|  | Reverse | GCGGGTACCTCAATCTTTTGCTGCTCTGTAAATC |
| *FLAG-SFA12a* | Forward | GCGGCTAGCATGAATCCAAATACACCCTTC |
|  | Reverse | GCGGGTACCTCAGTCTTTTGCTGCTCTG |
| *FLAG-SFA13a* | Forward | GCGGCTAGCATGAATCCCAATACACCTTTC |
|  | Reverse | GCGGGTACCTCAATCCTTGGCTGCTCTG |
| Calmodulin | Forward | CTGAAGCTGAACTTCAAG |
|  | Reverse | CAGAATGATGGTTTCTAAATGA |

Additional File 2: Supplemental Table 3 Predicted Positions of the coiled-coil domains in the SFA proteins and Accession Numbers.

| Gene Name | Accession Number | # AA | MW  kDa | Coiled-coil Domain 1 | Coiled-coil #AA | Coiled-coil Domain 2 | Coiled-coil #AA |
| --- | --- | --- | --- | --- | --- | --- | --- |
| SFA1a | GSPATP00025723001 | 272 | 31.81 | 30-58 | 29 | 161-226 | 66 |
| SFA1b | GSPATP00023935001 | 274 | 31.99 | 32-60 | 29 | 163-228 | 66 |
| SFA7a | GSPATP00036966001 | 251 | 29.27 | 31-63 | 33 | 175-240 | 66 |
| SFA7b | GSPATP00034214001 | 251 | 29.27 | 31-63 | 33 | 175-240 | 66 |
| SFA8a | GSPATP00032447001 | 257 | 29.72 | 34-119 | 86 | 213-243 | 31 |
| SFA8b | GSPAT00033989001 | 257 | 29.70 | 34-119 | 86 | 213-243 | 31 |
| SFA9 | GSPATT00011977001 | 255 | 29.55 | 32-117 | 86 | 211-241 | 31 |
| SFA10a | GSPATP00034929001 | 256 | 29.64 | 33-118 | 86 | 212-242 | 31 |
| SFA10b | GSPATP00028132001 | 256 | 29.61 | 33-118 | 86 | 212-242 | 31 |
| SFA10c | PTETP6800004001 | 256 | 29.57 | 33-118 | 86 | 212-242 | 31 |
| SFA10d | PTETP17800004001 | 256 | 29.48 | 33-118 | 86 | 212-242 | 31 |
| SFA11a | GSPATP00002032001 | 248 | 28.81 | 44-91 | 48 | 203-236 | 34 |
| SFA11b | GSPATP00017545001 | 248 | 28.81 | 44-91 | 48 | 203-236 | 34 |
| SFA12a | GSPATP00001334001 | 248 | 28.80 | 44-91 | 48 | 203-236 | 34 |
| SFA12b | GSPATP00002707001 | 248 | 28.80 | 44-91 | 48 | 203-236 | 34 |
| SFA12c | GSPATP00003161001 | 248 | 28.80 | 44-91 | 48 | 203-236 | 34 |
| SFA12d | GSPATP00004838001 | 248 | 28.74 | 44-91 | 48 | 203-236 | 34 |
| SFA13a | GSPATP00025686001 | 248 | 28.94 | 44-91 | 48 | 203-236 | 34 |
| SFA13b | GSPATP00030915001 | 248 | 28.98 | 44-91 | 48 | 203-236 | 34 |
| SFA13c | GSPATP00020721001 | 248 | 28.99 | 44-91 | 48 | 203-236 | 34 |
| SFA13d | GSPATP00021965001 | 248 | 29.15 | 44-91 | 48 | 203-236 | 34 |
| SFA6a | GSPATP00010780001 | 249 | 29.89 | 174-232 | 59 |  |  |
| SFA6b | GSPATP00014274001 | 249 | 29.91 | 174-232 | 59 |  |  |
| SFA6c | GSPATP00005889001 | 249 | 29.86 | 174-232 | 59 |  |  |
| SFA6d | GSPATP00014806001 | 249 | 29.99 | 174-232 | 59 |  |  |
| SFA2 | GSPATP00020957001 | 249 | 29.82 | 174-232 | 59 |  |  |
| SFA3 | GSPATP00022867001 | 249 | 29.89 | 174-232 | 59 |  |  |
| SFA4 | GSPATP00008626001 | 258 | 30.57 | 183-241 | 59 |  |  |
| SFA5a | GSPATP00026454001 | 249 | 29.93 | 174-232 | 59 |  |  |
| SFA5b | GSPATP00024123001 | 249 | 30.04 | 174-232 | 59 |  |  |
| SRL 1a | GSPATP00011812001 | 313 | 36.15 |  |  |  |  |
| SRL 1b | GSPATP00008129001 | 315 | 36.16 |  |  |  |  |
| SRL 2 | GSPATP00011715001 | 310 | 34.76 |  |  |  |  |
| SRL 3a | GSPATP00017369001 | 305 | 34.23 |  |  |  |  |
| SRL 3b | [GSPATP00019347001](https://paramecium.i2bc.paris-saclay.fr/cgi/browse/gene?uniquename=GSPATP00019347001) | 305 | 34.11 |  |  |  |  |
| SRL 3c | GSPATP00026761001 | 310 | 35.01 |  |  |  |  |
| SRL 4a | GSPATP00007006001 | 249 | 28.41 |  |  |  |  |
| SRL 4b | GSPATP00016662001 | 264 | 30.64 |  |  |  |  |
| SRL 5a | GSPATP00010287001 | 238 | 26.71 |  |  |  |  |
| SRL 5b | GSPATP00033449001 | 224 | 25.34 |  |  |  |  |
| SRL 5c | GSPATP00017294001 | 231 | 26.72 |  |  |  |  |

Additional File 2: Supplemental Table 4 SFA proteins found by LC-MS/MS of an Optiprep density faction from Control Cells (combined from 3 experiments).

C

|  | SFA protein | Total peptide sequences identified | Unique to the paralog |
| --- | --- | --- | --- |
| Paralog Group 1  (Structural Group 1) | SFA1a | 28 | 8 |
|  | SFA1b |  | 13 |
| Paralog Group 2-6  (Structural Group 5) | SFA2 | 60 | 5 |
|  | SFA3 |  | 1 |
|  | SFA4 |  | 5 |
|  | SFA5a |  | 2 |
|  | SFA5b |  | 4 |
|  | SFA6a |  | 0 |
|  | SFA6b |  | 0 |
|  | SFA6c |  | 3 |
|  | SFA6d |  | 3 |
| Paralog Group 7  (Structural Group 2) | SFA7a | 13 | 0 |
|  | SFA7b |  | 0 |
| Paralog Group 8-10  (Structural Group 3) | SFA8a | 35 | 1 |
|  | SFA8b |  | 0 |
|  | SFA9 |  | 3 |
|  | SFA10a |  | 0 |
|  | SFA10b |  | 3 |
|  | SFA10c |  | 0 |
|  | SFA10d |  | 2 |
| Paralog Group 11-13  (Structural Group 4) | SFA11a | 47 | 1 |
|  | SFA11b |  | 2 |
|  | SFA12a |  | 0 |
|  | SFA12b |  | 0 |
|  | SFA12c |  | 0 |
|  | SFA12d |  | 0 |
|  | SFA13a |  | 2 |
|  | SFA13b |  | 1 |
|  | SFA13c |  | 6 |
|  | SFA13d |  | 0 |

Additional File 2: Supplemental Table 5 SFA proteins found by LC-MS/MS, analysis of an Optiprep density faction from Structural Group 1 depleted cell (data combined from 3 experiments).

|  | SFA protein | Total peptide sequences identified | Unique  Peptide |
| --- | --- | --- | --- |
| Paralog Group 1  (Structural Group 1) | SFA1a | 0 | 0  0 |
|  | SFA1b |  |  |
| Paralog Group 2-6  (Structural Group 5) | SFA2 | 30 | 2 |
|  | SFA3 |  | 0 |
|  | SFA4 |  | 2 |
|  | SFA5a |  | 1 |
|  | SFA5b |  | 1 |
|  | SFA6a |  | 0 |
|  | SFA6b |  | 0 |
|  | SFA6c |  | 1 |
|  | SFA6d |  | 0 |
| Paralog Group 7  (Structural Group 2) | SFA7a | 7 | 0 |
|  | SFA7b |  | 0 |
| Paralog Group 8-10  (Structural Group 3) | SFA8a | 15 | 1 |
|  | SFA8b |  | 0 |
|  | SFA9 |  | 1 |
|  | SFA10a |  | 0 |
|  | SFA10b |  | 1 |
|  | SFA10c |  | 0 |
|  | SFA10d |  | 0 |
| Paralog Group 11-13  (Structural Group 4) | SFA11a | 27 | 1 |
|  | SFA11b |  | 1 |
|  | SFA12a |  | 0 |
|  | SFA12b |  | 0 |
|  | SFA12c |  | 0 |
|  | SFA12d |  | 0 |
|  | SFA13a |  | 2 |
|  | SFA13b |  | 1 |
|  | SFA13c |  | 2 |
|  | SFA13d |  | 0 |

Additional File 2: Supplemental Table 6 SFA proteins found by LC-MS/MS found by LC-MS/MS analysis of an Optiprep density faction from Structural Group 2 depleted cell (data combined from 3 experiments).

C

|  | SR protein | Total peptide sequences identified | Unique  Peptide |
| --- | --- | --- | --- |
| Paralog Group 1  (Structural Group 1) | SFA1a | 10 | 3 |
|  | SFA1b |  | 5 |
| Paralog Group 7  (Structural Group 2) | SFA7a | 0 | 0  0 |
|  | SFA7b |  |  |
| Paralog Group 2-6  (Structural Group 5) | SFA2 | 25 | 2 |
|  | SFA3 |  | 0 |
|  | SFA4 |  | 2 |
|  | SFA5a |  | 1 |
|  | SFA5b |  | 1 |
|  | SFA6a |  | 0 |
|  | SFA6b |  | 0 |
|  | SFA6c |  | 1 |
|  | SFA6d |  | 0 |
| Paralog Group 8-10  (Structural Group 3) | SFA8a | 12 | 1 |
|  | SFA8b |  | 0 |
|  | SFA9 |  | 1 |
|  | SFA10a |  | 0 |
|  | SFA10b |  | 1 |
|  | SFA10c |  | 0 |
|  | SFA10d |  | 0 |
| Paralog Group 11-13  (Structural Group 4) | SFA11a | 13 | 1 |
|  | SFA11b |  | 1 |
|  | SFA12a |  | 0 |
|  | SFA12b |  | 0 |
|  | SFA12c |  | 0 |
|  | SFA12d |  | 0 |
|  | SFA13a |  | 2 |
|  | SFA13b |  | 1 |
|  | SFA13c |  | 2 |
|  | SFA13d |  | 0 |
